# Parental fasting effects on offspring immune gene expression, epigenetic patterns, and gut microbiota in a species with male pregnancy (*Syngnathus typhle)*

**DOI:** 10.1186/s12915-026-02509-7

**Published:** 2026-01-14

**Authors:** Freya Adele Pappert, Nils Newrzella, Olivia Roth

**Affiliations:** Marine Evolutionary Biology, Zoological Institute, Christian-Albrechts-Universität Kiel, Am Botanischen Garten 1-9, 24118 Kiel, Germany

**Keywords:** Intermittent fasting, Syngnathid, Male pregnancy, Trans-generational plasticity

## Abstract

**Background:**

Intermittent fasting is widely promoted for its potential to improve health and extend lifespan, yet these benefits may come at a reproductive cost, potentially reducing parental fitness and offspring quality. While the inter- and transgenerational effects of fasting are increasingly studied, they remain poorly understood in species with unconventional reproductive roles. Investigating such effects in these systems is crucial, as the evolutionary trade-offs between somatic maintenance and reproductive investment may differ from those in species with conventional reproductive roles. In this study, we investigated the effects of intermittent fasting (IF) in a species with male pregnancy, *Syngnathus typhle*, by exposing mothers and fathers to either IF or ad libitum (AL) feeding before mating. Upon transfer of maternal eggs to paternal brood pouches, males remained on their assigned diets throughout pregnancy.

**Results:**

Offspring from all parental diet combinations AL(p) × AL(m), IF(p) × IF(m), AL(p) × IF(m), and IF(p) × AL(m) (with *p* = paternal and *m* = maternal) were analyzed at birth before first feeding alongside parents for morphology, immune and epigenetic candidate gene expression, and gut microbiota composition. Mothers under IF showed greater condition loss, leading to reduced offspring condition regardless of paternal diet, highlighting the importance of maternal provisioning through eggs. However, IF fathers exhibited increased immune activation and microbiome shifts that were mirrored in offspring, suggesting paternal priming via epigenetic and microbial inheritance. Offspring from mismatched parental diets showed disrupted immune-microbiome correlations, indicating that aligned parental cues support more stable offspring development.

**Conclusion:**

These findings highlight how parental nutritional history differentially shapes offspring phenotype through maternal and paternal pathways in a species with male pregnancy. Our results emphasize the value of studying species with diverse reproductive strategies and life histories to understand the full spectrum of trans-generational plasticity in nature.

**Supplementary Information:**

The online version contains supplementary material available at 10.1186/s12915-026-02509-7.

## Background

An organism’s traits are shaped not only by the genes it inherits but also by nongenetic influences from the environment (within-generational phenotypic plasticity, WGP), as well as the conditions the parents experienced (trans-generational plasticity, TGP) [1–3]. DNA-independent inheritance can occur through multiple, often overlapping, routes including reproductive elements such as eggs (and, to a lesser extent, sperm), as well as through parental care, feeding, or other forms of close contact. These routes transmit a diverse array of information: for instance, eggs may carry maternal hormones and metabolites [4–6], epigenetic marks shaped by parental life history [7, 8], immunological components such as antibodies (i.e., transgenerational immune priming — TGIP) [9–12], and even vertically inherited microbial communities [13–15]. In contrast, postnatal influences such as parental care or feeding may shape offspring phenotype through continued microbial transfer, behavioral imprinting, or immune priming [16]. Understanding the relative contributions of these factors to the expression of offspring phenotypes, and ultimately to their fitness, and how they change over time is crucial for predicting how organisms acclimatize and ultimately adapt to new and changing environments [1]. While plasticity, including TGP, can buffer organisms against stress in the short term, it can also generate phenotypic variation, some of which may confer a fitness advantage under novel conditions and thus be favored by selection, potentially buying time for genetic changes to follow [17, 18].

When mothers experience changes in their environment, this can alter offspring development via maternal effects, even in the absence of direct exposure during the offspring’s own early experiences [1]. For example, a child may grow up in a food-rich environment but exhibit traits shaped by maternal exposure to food scarcity (e.g., increased fat deposition). These traits could become beneficial if the child later encounters similar conditions. Since the mother has already navigated a famine or nutrient scarcity, she may pass down helpful cues to prepare her offspring for similar challenges [19]. In some cases, phenotypic adjustment in response to current environmental cues (WGP) may offer a better match to local conditions than relying on parental cues. However, when rapid or early-life responses are required, offspring may benefit from parental preconditioning via TGP [1]. A well-documented example of the phenomenon of gestational programming is the development of a “thrifty phenotype” in offspring of mothers who faced nutritional constraints during pregnancy. Offspring of mothers with intrauterine growth restriction (IUGR) often have low birth weight and are predisposed to metabolic diseases like obesity, hypertension, and diabetes later in life [20–23]. These offspring are programmed to efficiently acquire and use nutrients, a trait advantageous in environments with scarce food. However, in nutrient-rich settings, such acclimatization can lead to negative maladaptive outcomes like obesity [19]. Across some mammalian species, including rats and sheep [24–26], studies have shown similar links between maternal nutrient deficiencies during pregnancy and adult health issues in the offspring, further highlighting the broad impact of maternal experiences on offspring development [19].

Paternal effects are often overlooked in transgenerational research, partly because fathers do not gestate offspring and may have limited parental involvement. However, growing evidence suggests that fathers play a significant role in inheritance beyond genetics, particularly through sperm-mediated effects such as epigenetic modifications and small RNAs [27]. Advances in epigenetics are shifting the research landscape to recognize these paternal contributions. In parallel, genetic factors such as increased germline mutations associated with paternal age can also affect offspring health and lifespan by reducing sperm quality and integrity [28, 29]. Age-related changes can also affect resource allocation to ejaculate [30, 31], and in some species, offspring of older fathers have shorter lifespans and a higher incidence of chronic illnesses [32, 33], likely resulting from a combination of genetic and phenotypic changes associated with aging and cumulative environmental stress [34]. Beyond age, paternal diet can also shape offspring health, influencing metabolic function and early developmental outcomes [35]. Studies have shown that exposure to a high-fat diet before mating can alter offspring traits, including body weight and metabolic parameters, though these effects vary across species [36, 37]. These findings underscore the importance of considering paternal trans-generational plastic effects in shaping offspring development [35].

Although parental effects have been studied across diverse taxa, including insects and fish [9, 38, 39], many models of trans-generational plasticity focus on species with conventional reproductive roles, where females typically both gestate and provide the majority of parental care. As a result, we still lack a clear understanding of how both parents’ environmental experiences — before and during reproduction — contribute to offspring phenotype, particularly in species with unconventional reproductive strategies. Teleost fish exhibit a remarkable range of variation in parental care strategies. Among those species that do exhibit parental care, approximately half provide exclusive paternal care [40, 41]. In particular, the Syngnathidae family is renowned for its exceptionally dedicated male parental care, having evolved a range of male brooding forms [42, 43]. These adaptations span from the simple attachment of eggs to the male’s ventral surface (e.g., *Nerophis ophidion*) to the development of highly specialized brooding structures (e.g., *Hippocampus erectus*). These brooding structures are analogous to those found in eutherian mammals, and they can provide the developing embryos with protection, nutrients, oxygen, and immunological components [9, 44–47]. Furthermore, they can mediate transgenerational effects in response to environmental conditions such as salinity levels affecting offspring gene expression and survival [48] and shape the external juvenile microbiome through paternal provisioning [49]. Remarkably, the phenomenon of male pregnancy has its origins in a single evolutionary event, which then gave rise to approximately 300 distinct species of syngnathid [42]. The Syngnathidae family can be found in a broad range of temperate and tropical environments, including rocky and coral reefs, mangrove forests, seagrass beds, estuaries, and rivers [42].

Species like the *broadnosed pipefish* (*Syngnathus* *typhle*), with their reversed reproductive roles and male pregnancy, offer a unique opportunity to disentangle the contributions of maternal egg production from the physiological changes associated with paternal pregnancy (e.g., body reshaping and energy allocation) [45]. In this pipefish species, females typically produce, on average, more eggs than males can brood [50, 51], making males the more reproductively constrained sex [52]. Due to their polygamous mating system and the males’ limited reproductive capacity, males become the more choosy sex, often preferring larger females, while females exhibit ornamentation and actively court males [50, 52–55]. This species’ nonconventional mating system and *male pregnancy* make it an *ideal model* for studying how *parental effects* and *resource allocation* are shaped by sex-specific reproductive strategies. It allows for a more nuanced understanding of *how males and females may influence offspring development differently*, especially when disentangling *gestational vs. sex-specific effects* on offspring phenotype [56–58].

In this study, we examined the influence of dietary restriction on *Syngnathus typhle* by subjecting both males and females to intermittent fasting (IF) for a month prior to reproduction. Specifically, the experimental conditions included a twice-daily feeding schedule with live food for the ad libitum (AL) condition or IF with a feeding day followed by two fasting days. Individuals were then mated following various dietary combinations, with paternal (p) and maternal (m) combinations, hereafter abbreviated as AL(p) × AL(m), IF(p) × IF(m), AL(p) × IF(m), and IF(p) × AL (m). The IF treatment was continued for males throughout their pregnancy. After offspring were born, the young were sacrificed before first feeding, alongside their parents, to assess morphological characteristics, immune- and epigenetic-related gene expression, and gut microbiota composition through 16S rRNA sequencing. We hypothesized that offspring from IF parents would be morphologically smaller than those from AL parents, due to limited parental resource availability. Because males gestate the offspring in *Syngnathus typhle*, we expect paternal diet to have a stronger influence on offspring traits. In particular, we expected that the offspring’s gut microbiota and immune-related gene expression would reflect paternal feeding conditions, consistent with transgenerational plasticity mediated by male pregnancy.

Our findings open new avenues to explore how parental diet and sex-specific reproductive roles influence offspring development, particularly in species with reversed reproductive roles and male pregnancy. This study provides critical insights into transgenerational effects, gestational influences, and the role of the microbiome in offspring health while challenging existing paradigms by considering both maternal and paternal effects in shaping offspring phenotype.

## Results

### Intermittent fasting impacts parental and offspring condition

The cross-mating combinations resulted in 18 successful families, and this included 5 pregnant males in both AL(p) × AL(m) and IF(p) × IF(m) groups and 4 for the AL(p) × IF(m) and IF(p) × AL(m) groups. In the remaining 14 cases, mating was unsuccessful due to either male rejection or early loss of eggs. Specifically, in the AL(p) × AL(m) group, two males discarded eggs within a day, and one absorbed the few received; in IF(p) × IF(m), one male discarded the eggs, and two refused to mate; in AL(p) × IF(m), two males absorbed eggs, and two did not engage in mating; and in IF(p) × AL(m), one male absorbed the eggs, and three did not mate. Morphological measurements, including length, weight, and fat content, were calculated for the successfully mated individuals, totaling nine males and nine females per treatment (AL or IF), and then weight and length for their offspring.

There was no significant difference in initial body length between any of the randomly assigned males and females at the start of the experiment (Additional file 1: Fig. S3a, Additional file 2: Sheet A). Similarly, at the end of the experiment, final body lengths did not significantly differ between AL and IF groups within either sex (Additional file 1: Fig. S3a, Additional file 2: Sheet A). However, when considering growth over the treatment period, both AL males and females showed greater increases in body length compared to their IF-treated counterparts. Using the median and standard error, AL males grew from 12 cm ± 0.44 to 13.5 cm ± 0.43 over the 2-month diet period (*p* < 0.0001) and AL females from 12.6 cm ± 0.89 to 13.4 cm ± 0.79 over 1 month (*p* < 0.0001). In contrast, IF males showed a more modest increase from 12.5 cm ± 0.76 to 13.2 cm ± 0.78 and IF females from 12 cm ± 0.93 to 12.5 cm ± 0.87 (both *p* < 0.02) (Additional file 3 and Additional file 2: Sheet A). A similar pattern was observed for body weight (Fig. S3b). AL males increased from 0.62 g ± 0.07 to 0.94 g ± 0.09, while IF males showed a smaller change from 0.88 g ± 0.14 to 0.93 g ± 0.17. AL females grew from 0.84 g ± 0.24 to 0.89 g ± 0.19, whereas IF females showed a slight decline from 0.73 g ± 0.27 to 0.66 g ± 0.23. There was no significant difference between groups at the start of the experiment, but AL individuals showed a greater weight increase over the treatment period (*p* < 0.02), while IF individuals exhibited statistically insignificant weight changes (Additional file 3 and Additional file 2: Sheet A).

**Fig. 1:**
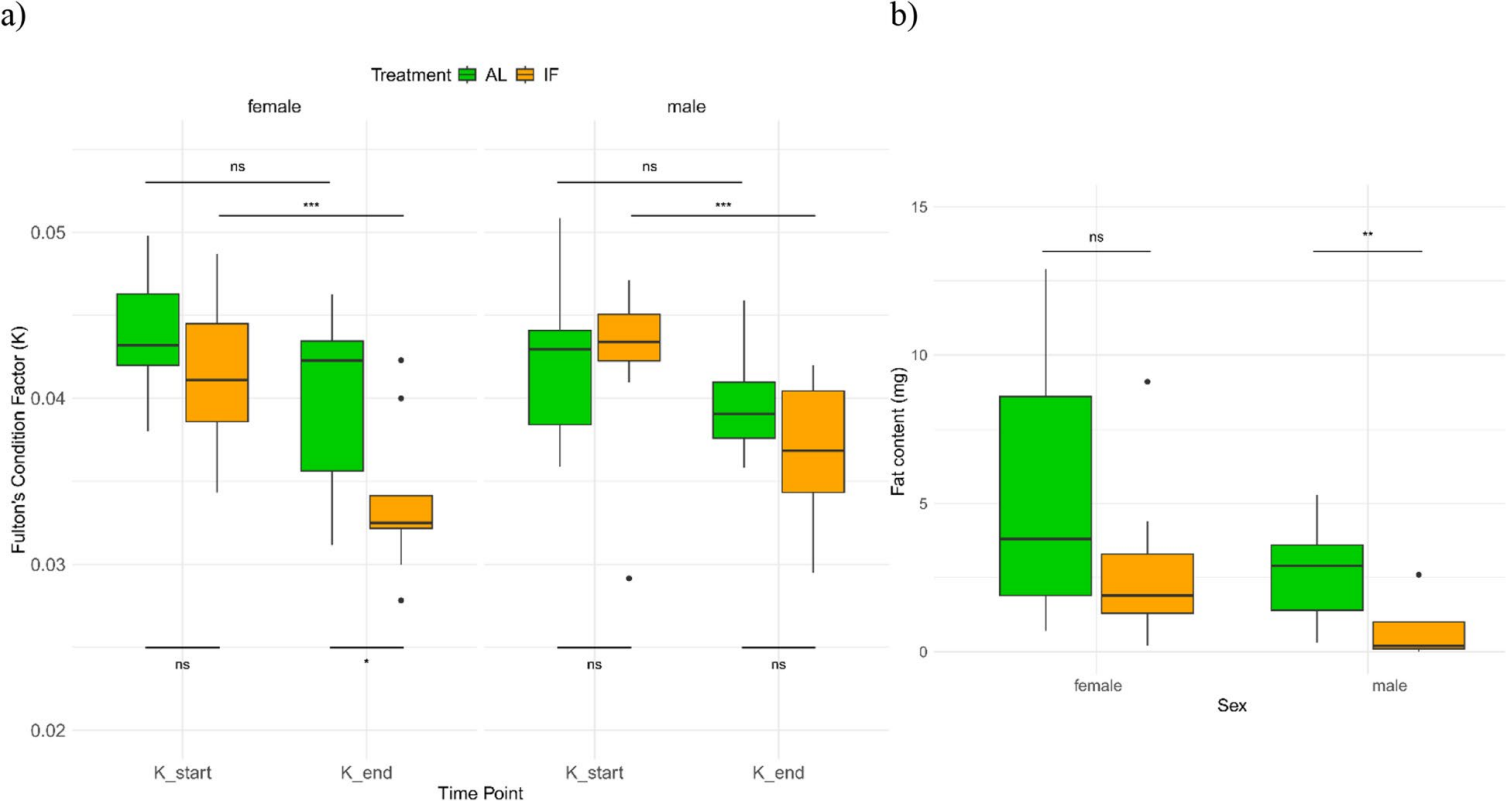
Condition index and fat content of *S. typhle* parents. **a** The plot displays Fulton’s condition factor (weight/length^³^) for sex and dietary treatment, over time (*x*-axis). AL individuals are represented in green, and IF individuals are in orange. Two-way ANOVA results comparing treatment groups for both time points and sexes were not significant (ns). Significant differences are denoted as *p < 0.05, **p < 0.001, and ***p < 0.0001. **b** Total body fat content (mg) of the pipefish parents. Females on the left had no significant difference in body fat content between AL and IF, while male pipefish were significantly different

In addition, we also assessed changes in overall body condition (Fulton’s condition factor). We found a significant overall effect of dietary treatment on the condition index at the end of the experiment (ANOVA, *p* < 0.002; Fig. 1a). Post hoc comparisons revealed that only females from the AL and IF groups differed significantly (Tukey’s HSD, *p* < 0.03). Furthermore, linear mixed model analyses showed a significant decline in the condition index over the fasting period for both IF females and males (*p* < 0.0001; Additional file 2: Sheet A), reflecting the impact of food restriction. In contrast, AL individuals maintained a relatively stable condition index throughout the experimental period, despite some variation. For total body fat content in the parent pipefish, no significant treatment effect was observed between IF and AL females. However, there was a significant difference for males (*p* < 0.01, Fig. 1b; Additional file 2: Sheet A).

**Fig. 2:**
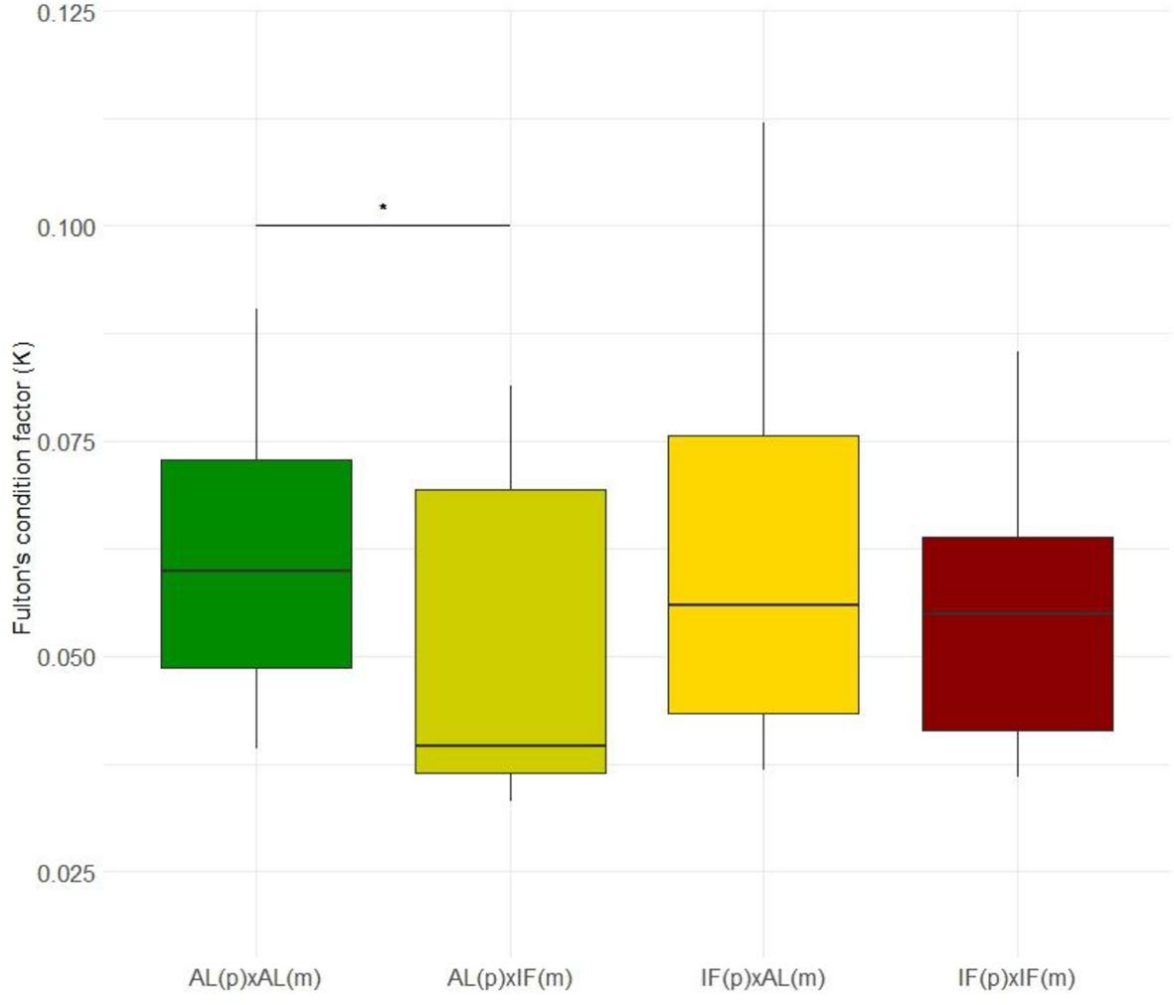
Offspring physiological condition at birth. Plot illustrates Fulton’s condition factor for offspring at birth across different parental dietary treatment groups: dark green for AL(p)×AL(m), dark yellow for AL(p)×IF(m), yellow for IF(p)×AL(m), and red for IF(p)×IF(m). Asterisk indicates the only significant pairwise comparison detected: AL(p)×IF(m) offspring has a slightly lower K than AL(p)×AL(m) (Dunn test, *p* < 0.05)

A total of 5 juveniles per breeding pair were collected after the males gave birth: 20 each for the AL(p) × AL(m) and IF(p) × AL(m) groups, 25 for the IF(p) × IF(m) group, and 16 juveniles for the AL(p) × IF(m) pairing. Gestation length did not differ between treatment groups: AL(p) × AL(m) 21.45 ± 0.27 days, IF(p) × IF(m) 22 ± 0.34 days, AL(p) × IF(m) 23.7 ± 0.7 days, and IF(p) × AL(m) 22 ± 0.5 days. Furthermore, the mean offspring length did not vary substantially across treatments, with AL(p) × AL(m) producing offspring with an average length of 20.97 ± 0.56 mm, IF(p) × IF(m) at 21.37 ± 0.55 mm, AL(p) × IF(m) at 23.21 ± 0.86 mm, and IF(p) × AL(m) at 21.84 ± 0.73 mm (Additional files 2 and 3). Offspring weight was, however, significantly different between groups (*p* < 0.05), with AL(p) × AL(m) offspring averaging 5.49 ± 0.20 mg, IF(p) × IF(m) at 5.12 ± 0.18 mg, AL(p) × IF(m) at 5.83 ± 0.23 mg, and IF(p) × AL(m) at 5.89 ± 0.27 mg. After adjusting for multiple comparisons using Tukey’s HSD, none of the pairwise comparisons for weight was significant, with only the IF(p) × IF(m) showing a trend toward being lighter than IF(p) × AL(m) (Additional file 2). When testing for Fulton’s factor, we could not find a strong parental dietary effect on offspring body condition and only a slightly lower K for offspring from AL(p) × IF(m) parents compared to AL(p) × AL(m) (Dunn test, *p* < 0.05, Additional file 2: Sheet A) (Fig. 2).

### Intermittent fasting drives gene expression shifts in fathers and their offspring

To gain an overview of gene expression patterns across all samples and assess how variation was structured in the dataset, we first conducted a principal component analysis (PCA) using ΔCt values for both parents and offspring combined. This approach allowed us to evaluate whether treatment effects might be stronger than differences related to generation or sex. In the combined dataset, principal component 1 (PC1) explained 34.3% of the variance, primarily capturing the separation between offspring and parental samples, along with some plate effects (Additional file 1: Fig. S5a and b and Additional file 2: Sheet B), while PC3 showed a marginal association with treatment (*p* = 0.058, Additional file 2). Given this clear generational distinction, subsequent analyses were conducted separately to disentangle treatment effects within each group. When focusing solely on offspring data, the association of PC3 with treatment effects strengthened (*p* < 0.02; Additional file 2: Sheet B), indicating a clearer effect of parental dietary treatment on offspring gene expression. Post hoc pairwise comparisons (*adonis2*) showed a stronger difference between specific treatment groups, notably between IF(p) × IF(m) and AL(p) × IF(m) (*p* = 0.05) (Fig. 4a and Additional file 2: Sheet B).

**Fig. 3:**
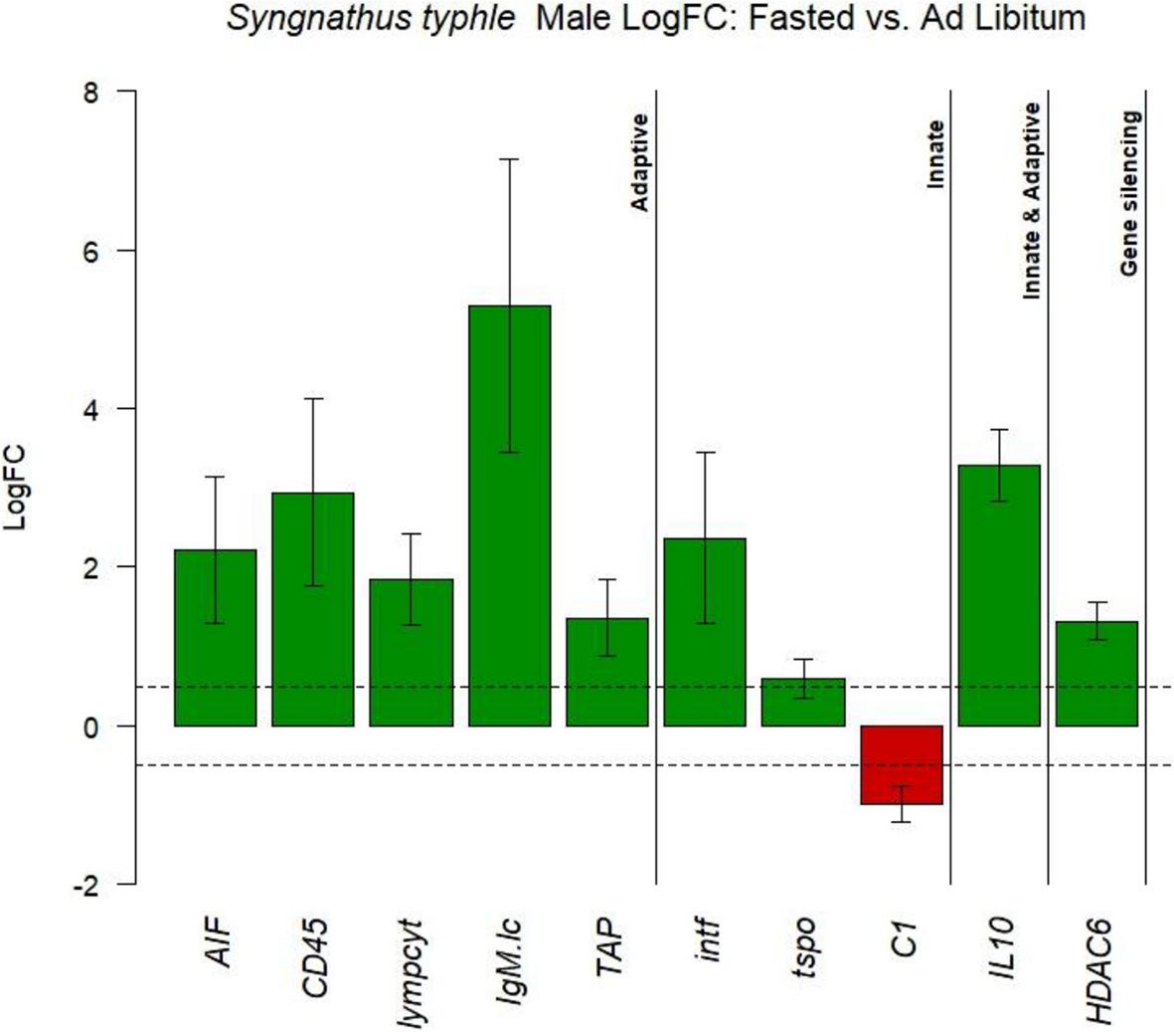
Bar plot of LogFC values for male *Syngnathus typhle* fathers, comparing IF to AL feeding as a control. The bar plot displays only those genes that were significant (*p* < 0.05) in the linear mixed model (LMM) analysis (Additional file 2) and met the LogFC threshold of ±0.5. Genes upregulated in fasted males are shown in green, while downregulated genes are in red. The genes are categorized into functional groups, including immune system categories (adaptive, innate, innate & adaptive) and gene regulatory groups (e.g., gene silencing). Of the 10 genes depicted, 9 are upregulated in fasted males relative to controls, with *C1* being the sole gene downregulated

Further analysis of individual gene expression responses to IF treatment in parents revealed varied effects in both sexes. In females, *DnMt3A* was the only gene with a significant response to fasting, reaching a log-fold change (LogFC) of 0.688 (LMM, *p* < 0.05; Additional file 2: Sheet C). In males, gene expression responses were more pronounced, particularly in immune-related pathways. Several markers of adaptive immunity — including *AIF*, *CD45*, *lymphocyt*, *IgM.lc*, and *TAP*, were significantly upregulated in response to IF. Likewise, innate immunity genes *intf* and *tspo* also showed significant upregulation under IF treatment (Fig. 3). Only one gene involved in both innate and adaptive immunity, *IL-10*, was significantly upregulated (Fig. 3). Among complement components, *C1* was the only gene downregulated by IF (Fig. 3). Additionally, the regulatory gene *HDAC6* was also upregulated, pointing to a broader immunomodulatory effect of intermittent fasting in males.

In pipefish offspring, several genes exhibited significant expression changes based on the parental treatment combinations, with AL(p) × AL(m) serving as the control. Within the adaptive immune response, *AIF* was just significantly upregulated in the IF(p) × AL(m) group (*LogFC* = 0.62, *p* = 0.05), while *bcell.rap31* was significantly downregulated in IF(p) × IF(m) offspring (*LogFC* = − 0.24, *p* = 0.03). For innate immune genes*, lectpt1* was strongly upregulated in the IF(p) × IF(m) group (*LogFC* = 2.86, *p* = 0.03), and *lectpt2* showed substantial upregulation in both AL(p) × IF(m) (*LogFC* = 5.36, *p* < 0.001) and IF(p) × IF(m) offspring (*LogFC* = 2.79, *p* = 0.03). The stress-related gene *hsp60* and *Intf* both showed nonsignificant trends toward downregulation in the IF(p) × AL(m) group (*LogFC* = − 0.39, *p* = 0.06, and *LogFC* = − 0.03, *p* = 0.06, respectively). Additionally, *ik.cytokine*, a gene implicated in both innate and adaptive immunity, was also downregulated in IF(p) × AL(m) offspring (*LogFC* = − 0.16, *p* = 0.06).

Regulatory genes were also significantly affected by parental diet treatments, particularly in offspring of IF-treated fathers (IF(p) × AL(m)), including *HDAC3* (*LogFC* = − 0.29, *p* = 0.03), *BROMO* (*LogFC* = − 0.26, *p* = 0.04), *JmjC_PHD* (*LogFC* = − 0.54, *p* = 0.02), *MYST1* (*LogFC* = − 0.49, *p* < 0.01), and *NO66* (*LogFC* = − 0.36, *p* < 0.001). For both AL(p) × IF(m) and IF(p) × IF(m) groups, *MYST1* was also downregulated (*LogFC* = − 0.39, *p* = 0.01; *LogFC* = − 0.36, *p* = 0.02), and *NO66* was equivalently so, although not always significantly (*LogFC* = − 0.26, *p* = 0.08; *LogFC* = − 0.34, *p* = 0.01, respectively). However, it is worth noting that not all of these genes met the ± 0.5 LogFC threshold (Additional file 2: Sheet D), suggesting more moderate regulatory shifts in response to parental diet.

**Fig. 4:**
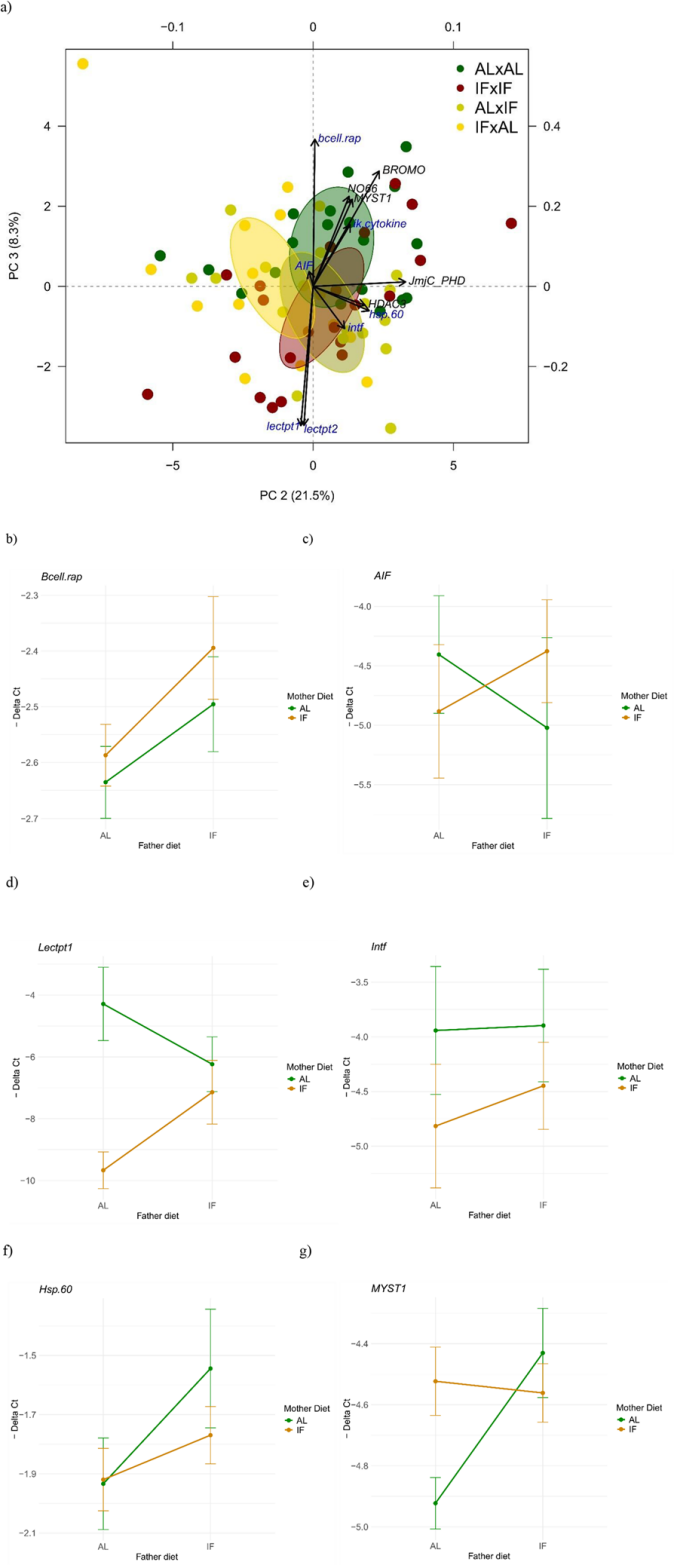
Parental dietary treatment effects on offspring gene expression. **a** PCA plot of differentially expressed genes in offspring, based on parental dietary combinations (dark green for AL(p)×AL(m), red for IF(p)×IF(m), dark yellow for AL(p)×IF(m), and yellow for IF(p)×AL(m)).The *x*-axis and *y*-axis represent PC2 and PC3, explaining 21.5% and 8.3% of the variance, respectively. Secondary axes display the gene loading coordinates, with arrow lengths scaled to reflect each gene’s contribution to these principal components. This plot highlights only those genes identified as differentially expressed through a linear mixed model (LMM) analysis (Additional file 2: Sheet C). Genes categorized as regulatory are labelled in black, while immunological genes are in dark blue. Plots b, c, d, e, f, and g show interaction plots for significantly differentially expressed genes in *S. typhle* offspring, including *Bcell.rap31* (**b**), *AIF* (**c**), *Lectpt1* (**d**), *Intf* (**e**), *Hsp.60* (**f**), and *MYST1* (**g**). Additional genes, including *Lectpt2*, *Ik.cytokine*, *BROMO*, *HDAC3*, *NO66*, and *JmjC-PHD*, are detailed in the supplementary material (Additional file 1: Fig. S5a, b, c, d, e, f). In these plots, the *x*-axis represents the father’s diet, while color coding (green for ad libitum and orange for intermittent fasting) indicates the mother’s diet. The *y*-axis displays the negative Delta Ct values, reflecting the directionality of gene expression. Each plot illustrates the interactions between parental dietary treatments and their effects on offspring gene expression.

To further elucidate the influence of parental dietary combinations on offspring gene expression, we conducted an interaction analysis comparing − ΔCt values between AL and IF diets for both fathers and mothers for the most significant genes (Fig. 4b, c, d, e, f, g).

When examining *Bcell.rap31* expression, the father’s diet played a dominant role: offspring exhibited low expression levels when the father was on an AL diet, regardless of the mother’s diet (Fig. 4b). However, if the father was IF, *Bcell.rap31* expression in offspring increased significantly, suggesting a paternally driven effect on this adaptive immune gene. Also *Hsp.60* demonstrated a more paternally driven influence, with expression levels consistently lower when the father was on an AL diet, irrespective of the mother’s diet (Fig. 4f). Offspring expression of *Hsp.60* increased only when the father was on an IF diet, underscoring the father’s dietary intake as the main driver.

Patterns in the innate immune genes *Lectpt1* and *Lectpt2* mirrored each other closely (Fig. 4c and Additional file 1: Fig. S5a). Expression was highest in offspring when both parents were on an AL diet, while offspring from an IF mother with an AL father showed the lowest levels. Interestingly, mixed diet pairs (AL(p) x IF(m) and IF(p) × AL(m)) resulted in intermediate expression levels, suggesting that parental diet mismatch modulates gene expression toward a balance between the extremes.

For *Intf*, the maternal diet emerged as the primary influencer: offspring exhibited higher expression when the mother was on an AL diet, with expression levels dropping under an IF maternal diet (Fig. 4e). This trend persisted regardless of the father’s diet, indicating a maternal dominance in regulating *Intf* expression.

In the case of *Ik.cytokine*, parental IF diets synergistically amplified expression, with the highest levels observed when both parents were on an IF diet (Additional file 1: Fig. S5b). Even when only one parent was on an IF diet, *Ik.cytokine* expression increased, suggesting that any exposure to IF in either parent potentiated this gene’s expression, with dual-IF parental treatment yielding the greatest effect.

A similar interaction was observed for *AIF*, which showed elevated expression when both parents shared the same diet, either AL or IF (Fig. 4c). However, expression of *AIF* decreased when parental diets differed, hinting at a synchrony effect where parental dietary alignment might maximize expression levels.

Interestingly, all regulatory genes followed a similar diet-dependent expression pattern, regardless of whether they function as activators (*BROMO* and *MYST1*) or repressors (*NO66*, *JmjC-PHD*, and *HDAC3*). In all cases, offspring from AL(p) × AL(m) parents showed the lowest expression levels, while those from an IF father and AL mother consistently exhibited the highest (Additional file 1: Fig. S5c, d, e, f). The mother’s fasting status appeared to have minimal impact, causing only slight fluctuations when she was IF, suggesting a significant paternal influence on regulatory gene expression in offspring.

### Fasting affects microbial composition of fathers and offspring

**Fig. 5:**
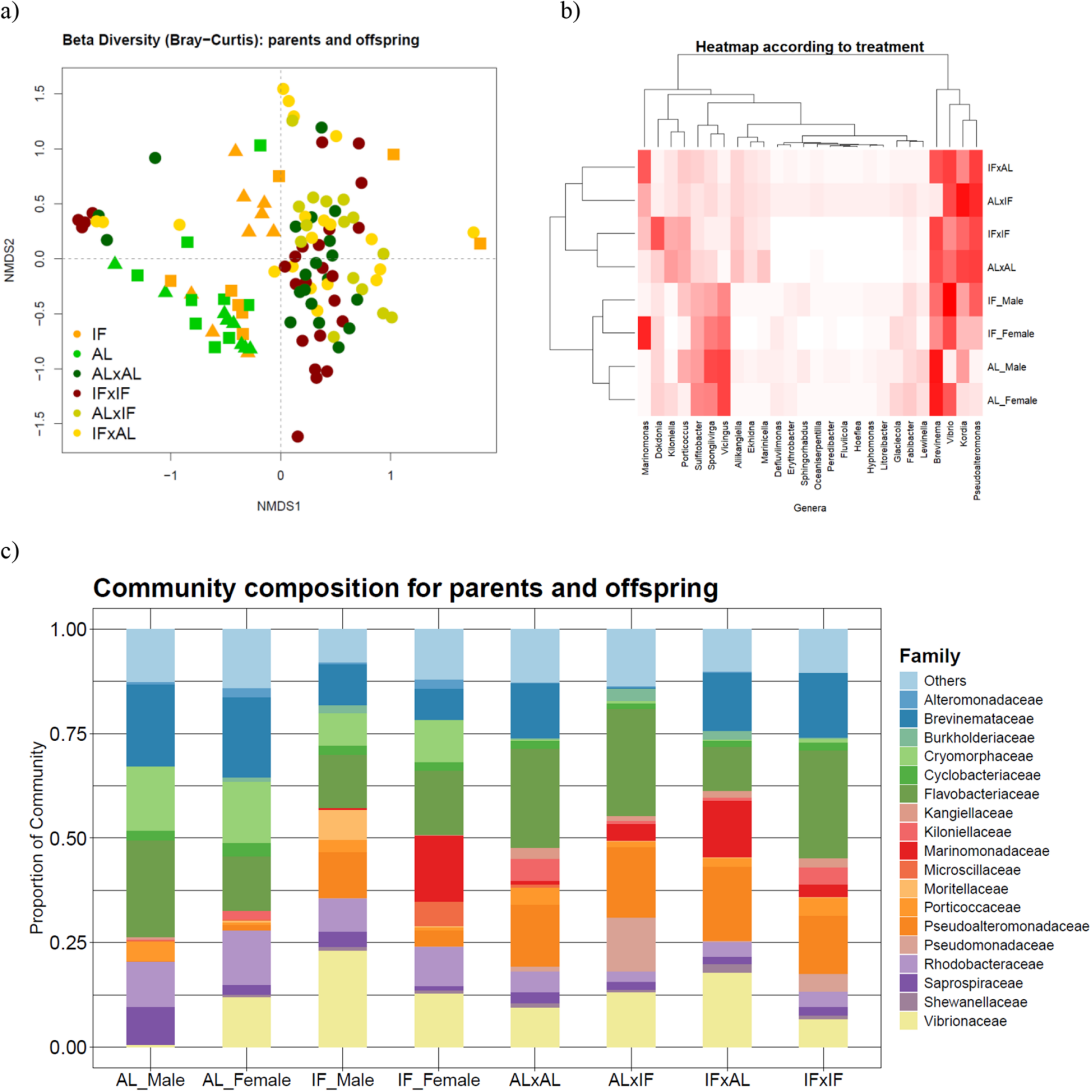
Parental diet-dependent variation in gut microbiota beta diversity and taxonomic composition. **a** Bray-Curtis NMDS plot illustrating bacterial community composition in pipefish parents and their offspring. Points are colored by diet: fasting parents are shown in orange and ad libitum parents in light green. Offspring color-coding reflects parental diet combinations, with dark green for offspring from both AL parents, dark red for offspring from both fasting parents, olive green for offspring of an AL male and IF female, and yellow for offspring of IF male and AL female. Parental sex is denoted by symbols: an upward triangle represents males, and a square indicates females. NMDS1 shows clear separation between offspring and parents, highlighting distinct bacterial community compositions across generations. **b** Heatmap of the most significant (*p* < 0.01) taxa driving Bray-Curtis beta-diversity patterns. Taxa abundance is shown by red intensity, with darker shades indicating higher relative abundance. The *y*-axis dendrogram clusters samples based on treatment groups (e.g., IF(p)×AL(m), AL(p)×IF(m)), while the *x*-axis dendrogram clusters taxa by similarity. **c** Bar plots showing the proportional composition of bacterial communities at the family level, highlighting the 20 most abundant taxa across all groups

Microbial alpha-diversity metrics revealed no significant differences across the groups — neither between parents and offspring nor between treatment groups (AL vs. IF) (Additional file 2: Sheet E). This suggests that within-group microbial richness and evenness remained stable. In contrast, beta-diversity analysis showed significant differences across groups with Bray–Curtis (PERMANOVA, *p* < 0.001). Pairwise comparisons revealed that the strongest differences occurred between parents and offspring (Additional file 2: Sheet F), as illustrated in the NMDS plot (Fig. 5), where NMDS1 clearly separates the generational differences. Nonetheless, when incorporating phylogenetic distances with UniFrac analysis, we found no clear differences across sex, diet, or generation (Additional file 1: Fig. S6; Additional file 2: Sheet F), indicating that the relative abundances of specific taxa may differ but the underlying bacterial lineages present are largely shared.

Further inspection within the adult groups showed no significant differences in beta diversity between AL males and AL females or between AL females and IF females (Additional file 2: Sheet F). However, a significant difference emerged between AL males and IF males (*p* = 0.02), indicating that fasting influenced microbial community composition in male pipefish but not in females. This distinction is also reflected in Fig. 5a, where IF males tend to cluster toward the upper region, while other adults are generally concentrated toward the bottom left. Among offspring groups, the most pronounced differences in microbial composition were between the AL(p) × IF(m) and other offspring pairings (*p* < 0.05).

The bacterial genera driving the observed separation between parental and offspring gut microbiomes are illustrated in Fig. 5b (Additional file 2: Sheet F). Notably, certain genera — such as Vicingus (Cryophormacea), Spongiivirga (Flavobacteriaceae), and Sulfitobacter (Rhodobacteraceae) — were consistently more abundant in parents compared with offspring (Fig. 5b), reinforcing the distinct microbial profiles identified by the Bray–Curtis beta-diversity analysis. For instance, *Vicingus* was present at higher relative abundance in adult samples (7–15%) but remained below 0.4% in offspring samples (Fig. 5c). Conversely, *Kordia*, *Pseudoalteromonas*, and* Aliikangiella* showed greater relative abundance in offspring, with *Pseudoalteromonas* appearing more prominently in offspring of fasting parents (Fig. 5b and c).

Within the parental groups, diet also shaped microbial composition, as evidenced by increased *Brevinema* in AL-fed parents and higher *Pseudoalteromonas* in IF parents (Fig. 5c). Among offspring, combinations of different parental diets (AL(p) × IF(m) or IF(p) × AL(m)) and similar ones clustered together (Fig. 5a and b), with example decreased relative abundances of *Dokdonia*, *Kiloniella*, and *Porticoccus* for IF(p) × AL(m) and AL(p) × IF(m).

**Fig. 6:**
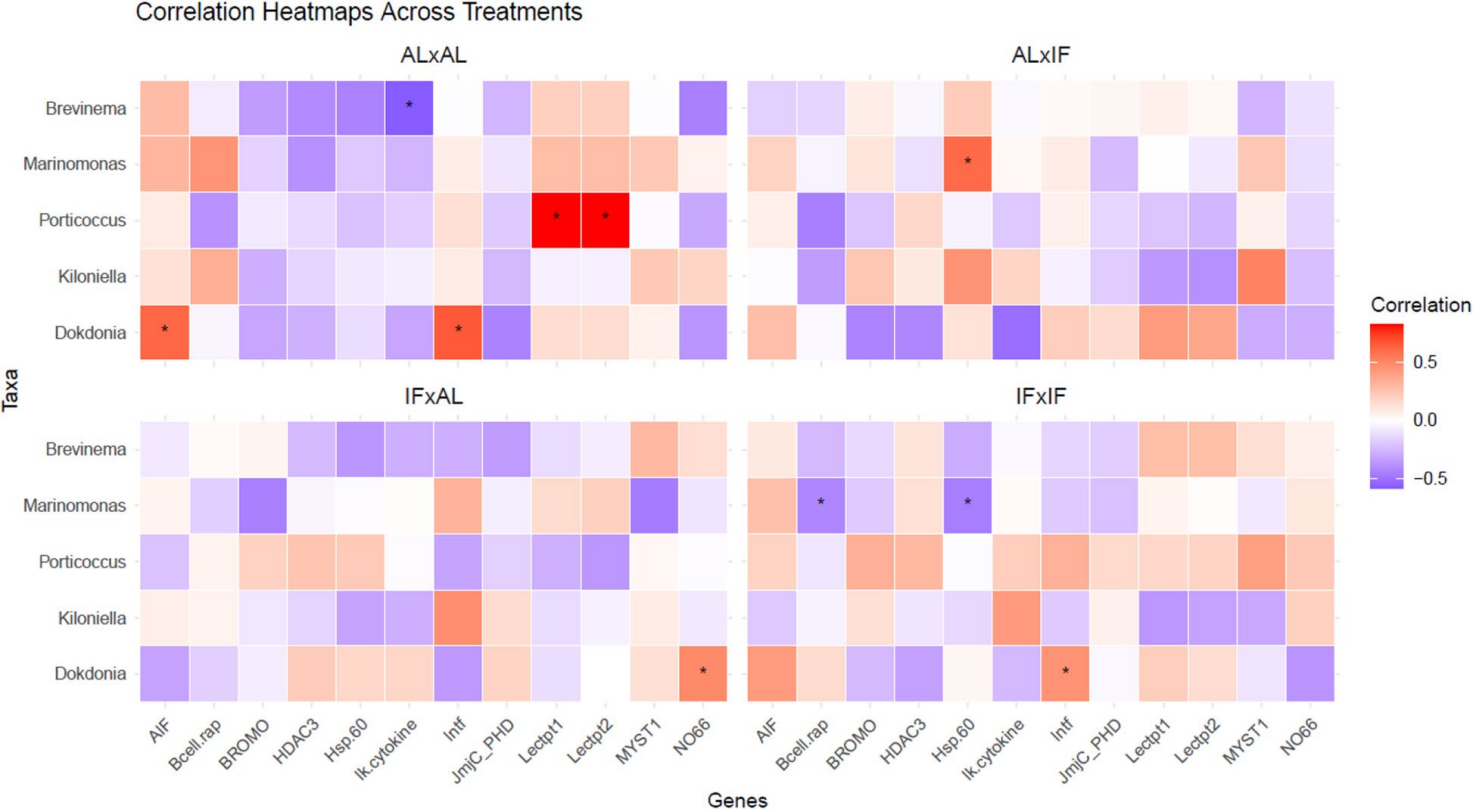
Spearman correlation heatmap depicting the relationships between significant differentially expressed genes and microbial taxa likely driving offspring treatment divergence for offspring from the four different parental dietary treatment groups: (AL(p) × AL(m), AL(p) × IF(m), IF(p) × AL(m), and IF(p) × IF(m)). The *x*-axis represents the genes, while the *y*-axis displays the microbial genera. Color intensity reflects the strength of the correlation, with dark blue indicating *r* < − 0.5 and dark red indicating *r* > 0.5. Asterisks (*) indicate correlations with uncorrected *p*-values < 0.05 from Spearman correlation tests. *p*-values are shown for exploratory purposes only; none of the correlations remained significant after false discovery rate (FDR) correction, indicating that these associations should be interpreted as hypothesis generating rather than confirmatory

The Spearman pairwise correlation analysis between the genera *Brevinema*, *Marinomonas*, *Porticoccus*, *Kiloniella*, and *Dokdonia* and the offspring genes previously identified as differentially expressed revealed a limited number of nominal associations prior to post hoc multiple-testing correction (Fig. 6). The strength and direction of these association patterns varied across dietary combinations, with more pronounced associations observed in AL(p) × AL(m) and IF(p) × IF(m) offspring compared to the mixed diet groups (Fig. 6). In offspring from the AL(p) × AL(m) parental dietary combination, several relatively higher-magnitude correlations were observed between specific microbial taxa and immune-related gene expression, including a negative association between *Brevinema* and *Ik.cytokine* (*p* < 0.05) and positive associations between *Dokdonia* and *AIF* and *Intf* (*p* < 0.05). *Porticoccus* was associated with higher expression of *Lectpt1* and *Lectpt2* in this group (*p* < 0.05). Although these associations were weaker and did not remain statistically significant in IF(p) × IF(m) offspring, the direction of the associations was similar. In mixed dietary groups (AL(p) × IF(m) and IF(p) × AL(m)), *Porticoccus* showed negative associations with *Lectpt1* and *Lectpt2*, though these correlations were also not statistically significant. *Dokdonia* was recurrently associated with immune-related genes, showing positive associations with *Intf* in both AL(p) × AL(m) and IF(p) × IF(m) offspring and with *NO66* in IF(p) × AL(m) (*p* < 0.05). *Marinomonas* displayed opposing association directions with *Hsp.60*, being positively associated in AL(p) × IF(m) offspring and negatively associated in IF(p) × IF(m) offspring (*p* < 0.05).

## Discussion

Shifting environmental conditions frequently require organisms to rely not only on immediate plastic responses but also on TGP, where survival often depends on the intergenerational transfer of biological experience [1]. This inherited knowledge, encoded in physiological and epigenetic marks, microbial communities, or behavioural cues, can improve offspring–environmental matching and potentially serve as evolutionary rescue [59]. The current understanding of how parents influence their offspring is predominantly based on conventional sex roles, where mothers bear the responsibility of both the production of costly gametes and pregnancy. However, nature has evolved a diverse array of reproductive strategies and parenting roles. Reproductive strategies might profoundly influence nongenetic inheritance, i.e., parental care might enhance the opportunity for sex-specific trans-generational transfer fostered by intimate contact of one parent with the offspring. The timepoint of transfer of parental experience, e.g., whether during the fusion of egg and sperm or, alternatively, during pre- or postnatal parental care, might additionally shape the outcome and role of nongenetic inheritance in the offspring’s life. To gain a comprehensive understanding of how parental experiences shape subsequent generations, we must expand our perspective beyond the familiar and explore systems where the functions of sex, care, and gestation are dissociated.

The broadnosed pipefish, with its reversed sex roles and male pregnancy, presents a rare opportunity to disentangle the effects of maternal provisioning from the physiological impacts of gestation [48, 57, 60]. This species’ exceptional reproductive strategy makes it an ideal model for exploring how sex-specific parental environmental stressors shape offspring phenotypes [48, 61, 62]. By leveraging this distinct reproductive system, our study aimed to disentangle the relative contributions of maternal provisioning and the paternal gestational environment to offspring development, offering novel insights into how sex-specific and gestational effects interact under nutritional stress. In the present study, we examined the influence of parental diet on offspring development by exposing both male and female *S. typhle* to either intermittent fasting (IF) or ad libitum (AL) feeding prior to mating and continuing the IF regime during male pregnancy. We then assessed morphological traits, immune and epigenetic gene expression, and gut microbiota composition in both parents and their offspring. While our design captures both maternal and paternal contributions, including paternal care via male pregnancy, it is possible that some of the observed offspring effects stem from early, pre-fertilization influences such as epigenetic modifications in gametes. Distinguishing between such pre- and post-fertilization mechanisms is a key future goal which requires comparative experimental approaches between closely related species along a parental care gradient. This study provides a first stepping stone toward disentangling the relative roles of gametic programming and parental provisioning in shaping offspring phenotype.

The IF regime imposed a clear energetic cost, as evidenced by reduced growth in length and weight (Additional file 1: Fig. S3), and declines in Fulton’s condition index (K) in both sexes over time (Fig. 1a). Notably, only IF females were in a weaker endpoint condition than their AL counterparts (*p* < 0.05), despite males also exhibiting fat loss (*p* < 0.01, Fig. 1b) and suppressed growth (Fig. 1a). This is particularly striking given that males were subjected to the IF regime for twice as long as females. Females showed no reduction in fat content (Fig. 1b), suggesting that the decline in K may reflect loss of lean tissue such as muscle. This female-specific sensitivity is striking, particularly given that males were fasted for twice as long. A similar pattern has been reported in *Hippocampus erectus*, where caloric restriction affected condition in females but not males, and was linked to female-biased metabolism and fat storage strategies [56]. Such sex-specific responses are conserved across taxa: in mice, for instance, males tend to preserve muscle and bone by mobilizing fat stores, while females resist fat loss and instead catabolize lean mass, often alongside reproductive suppression [63, 64]. Our findings suggest that female pipefish may similarly be more vulnerable to fasting, possibly due to sex-specific energy allocation strategies. Alternatively, males may have compensated for long-term restriction through physiological or behavioral adjustments.

Although offspring morphology was only marginally affected by parental dietary treatment — mean lengths across groups were fairly similar (~ 21–23 mm), and IF(p) × IF(m) offspring trended toward lower weight — we detected a significant difference in offspring condition (Fig. 2). Specifically, offspring from AL(p) × IF(m) crosses had a lower condition index compared to those from AL(p) × AL(m) crosses (Dunn test, *p* < 0.05; Fig. 2). Since juveniles were measured immediately after birth to capture parental and gestational effects before environmental variation could influence phenotype, it is possible that latent or delayed morphological effects could emerge later in development (e.g., in growth rate, behavior, or survival). Nonetheless, these findings indicate that maternal physiological state or egg provisioning plays a crucial role in shaping early juvenile condition, even in a species that relies exclusively on post-fertilization paternal care. A well-fed father (AL) was unable to fully buffer against the consequences of reduced maternal input (IF), while offspring from fasted fathers and well-fed mothers (IF(p) × AL(m)) showed no such deficit, even though paternal feeding conditions remained throughout pregnancy. This asymmetry highlights the central role of maternal investment in early development in fish [65, 66]. While males in *S. typhle* contribute significantly to gestation, egg quality remains a limiting factor [56, 62]. Our results suggest that maternal provisioning can more effectively compensate for suboptimal paternal condition than vice versa, possibly because females are able to prioritize and sustain ovarian function even under IF — drawing on somatic reserves at the expense of body condition — while incurring a lower energetic cost than males investing in pregnancy.

Principal component analysis of gene expression profiles showed that variation was predominantly structured by life stage (offspring vs. parents), with only subtle effects of dietary treatment emerging on PC3 (Fig. 4a, Additional file 1: Fig. S4a, b, c). In females, *DnMt3A* — a key regulator of de novo DNA methylation [67] — was the only gene to surpass both the significance threshold and a LogFC of 0.5 under IF treatment. All adult fish were sampled immediately after completing their respective reproductive investment (i.e., egg production in females, pregnancy in males) to capture gene expression signatures at the biologically relevant endpoint of parental allocation. The modest transcriptional response in females may reflect a combination of shorter IF exposure and sex-specific physiological strategies. Indeed, this limited change aligns with our phenotypic observations, where females exhibited reduced condition but no measurable fat loss, suggesting distinct energy mobilization and possibly constrained regulatory flexibility under dietary stress. In contrast, IF males exhibited a broader molecular shift with pronounced upregulation of genes associated with the adaptive immune response, including *AIF*, *CD45*, *TAP*, and *IgM.1c*, alongside elevated expression of anti-inflammatory cytokines (*IL-10* and *intf*) and a downregulation of the complement component *C1* (Fig. 3). This transcriptional profile is consistent with established immunological responses to intermittent fasting. IF is known to enhance autophagy, a cellular process that facilitates the clearance of intracellular pathogens and damaged components while reducing oxidative stress through decreased ROS levels and increased SIRT1 expression [68, 69]. Furthermore, the observed downregulation of *C1*, a component of the classical complement pathway, aligns with the shift toward an anti-inflammatory and possibly more immune-tolerant state under energetic constraint. The upregulation of *CD45* — a pan-leukocyte marker critical for T- and B-cell receptor signalling — further suggests that fasting stimulates immune cell viability and activity, possibly through autophagy-mediated pathways. In mammals, short-term intensive fasting has been shown to enhance the survival and function of CD45 + leukocytes by reducing apoptosis and promoting cytokine secretion [68, 70], a mechanism that may be evolutionarily conserved and similarly activated in *S. typhle*. These findings suggest that male pipefish adjust their immune expression more readily than females under nutritional stress, perhaps reflecting the physiological demands of male pregnancy. Under nutritional stress, this balance might shift — forcing the immune system to compensate more strongly to maintain both pouch health and self-protection. Reduced investment in growth or fat stores under IF allows reallocation of limited resources to immune defense, especially in pregnant males.

The upregulation of immune-related genes in IF males coincided with significant shifts in gut microbial composition, suggesting a potential link between microbial signals and host immune modulation. Specifically, IF males showed increased abundance of *Pseudoalteromonas*, a genus within Proteobacteria, also found to dominate in calorie-restricted seahorses [56]. Proteobacteria are known to support gut health by promoting epithelial integrity and facilitating colonization by strict anaerobes while also influencing mucosal immune responses [71]. In contrast, AL-fed males displayed higher levels of *Brevinema*, a genus previously associated with fish mucus [72] and notably implicated in paternal-specific microbial transmission in syngnathids [15]. This microbial shift may reflect not only differences in dietary input but also differences in immunological state, as *Brevinema* dominance in AL males was paralleled by reduced expression of immune genes compared with the more activated immune profile of IF males. Given that IF has been shown to enhance epithelial barrier function and modulate immune responses through microbiota-driven mechanisms, it is plausible that the microbial changes observed here contribute to the transcriptional immune shifts in IF males [68, 73]. Moreover, the reduction in *Brevinema* under IF may indicate a disruption in paternal microbial transfer potential, possibly linked to altered immune signalling or reduced capacity to host microbes during reproduction.

Gene expression analyses in pipefish offspring revealed nuanced yet meaningful immunological and regulatory responses to parental dietary history. These effects were not uniform across gene types or treatment combinations, but instead appeared to reflect parent-specific transmission of dietary stress signals, with IF(p) × AL(m) and IF(p) × IF(m) offspring showing the strongest expression shifts. The consistent upregulation of chromatin remodelling and transcriptional regulatory genes (e.g., *HDAC3*, *JmjC-PHD*, *MYST1*, *BROMO*, *NO66*; Additional file 1: Fig. S5c, d, e, f, g) in offspring of IF-fed males suggests that paternal nutritional stress may prime offspring for broader developmental plasticity or environmental responsiveness, perhaps via epigenetic reprogramming. This is consistent with findings in other taxa where paternal diet has been shown to influence offspring metabolism, behavior, and disease susceptibility through modifications such as DNA methylation and histone acetylation [35, 74, 75]. Although genome-wide epigenetic reprogramming occurs post-fertilization, certain epigenetic marks can be maintained across generations — particularly at imprinted loci or other regulatory hotspots — allowing paternal nutritional experiences to influence early developmental trajectories [74]. This could enable the transmission of anticipatory cues to offspring, potentially shaping their metabolic or immune responsiveness in environments predicted to be resource-limited. Additionally, both *Bcell.rap* — essential for B-cell development and proliferation [76] — and *Hsp60* — a mitochondrial stress-related gene [77] — showed increased expression in offspring when fathers were IF, regardless of maternal diet (Fig. 4b and f). *Hsp60* functions primarily as a mitochondrial chaperone but also contributes to broader cellular processes including cell proliferation, apoptosis, migration, and immune regulation [78]. The simultaneous upregulation of these genes suggests that paternal fasting may act as a cue, priming offspring for anticipated physiological stress by enhancing immune readiness. This points to a form of intergenerational plasticity, where paternal nutritional status modulates offspring physiology through inherited immunological components [9, 60].

Rather than enhancing offspring immune preparedness, maternal IF appeared to dampen innate immune gene expression in the next generation. Offspring of IF-fed mothers showed a consistent trend toward lower expression of key innate immune genes such as *lectpt1*, *lectpt2*, and *intf* (Fig. 4d and e, Additional file 1: Fig. S5a). Additionally, *ik.cytokine* was upregulated in these same offspring (Additional file 1: Fig. S5b), a gene associated with the suppression of IFN gamma, potentially through downregulation of MHC class I expression [79]. This pattern suggests that maternal fasting may constrain investment in early immune programming, possibly due to energetic limitations during oogenesis. This interpretation is supported by other findings in *S. typhle* showing that environmental stressors like elevated temperatures can erase transgenerational immune priming, likely reflecting trade-offs between survival and immune investment when resources are limited [48, 57]. Our results may reflect a similar mechanism, where maternal stress cues lead to reduced immune provisioning as an adaptive reallocation of resources under caloric constraint.

Parental dietary treatment not only shaped offspring gene expression but also influenced their gut microbial composition. Taxonomically, offspring microbiomes differed markedly from those of adults, with genera like *Vicingus*, *Spongiivirga*, and *Sulfitobacter* (taxa associated with marine environments [80, 81]) abundant in parents but nearly absent in offspring (Additional file 1: Fig. 5b and c). It is crucial to highlight that these differences may not solely reflect parental dietary influences, juveniles naturally consume a very different diet at birth (e.g., copepods), whereas adults feed on larger invertebrates [82]. As such, developmental dietary divergence likely contributes to these microbial shifts. Conversely, *Pseudoalteromonas*, *Kordia*, and *Aliikangiella* were more prevalent in juveniles, with *Pseudoalteromonas* particularly enriched in offspring of fasting parents, mirroring IF parents and suggesting a microbial legacy of parental nutritional state (Fig. 5b and c). This genus includes marine species known for a wide range of functions, such as antibacterial, algicidal, and antiviral activities, as well as specific strains that prevent the settlement of common fouling organisms [83].

Nonetheless, PCA and Bray–Curtis beta-diversity analyses revealed that offspring microbiomes clustered by parental diet combinations (Fig. 5a and b). Offspring from mixed-diet crosses (AL × IF, IF × AL) harbored increased levels of *Marinomonas*, whereas those from matched parental diets (AL × AL and IF × IF) showed higher relative abundances of *Dokdonia*, *Kiloniella*, and *Porticoccus*. Correlation analysis (Fig. 6) further indicated that *Marinomonas* was positively associated with the stress-responsive gene *Hsp60* only in AL × IF offspring but negatively associated in both matched-diet groups. Meanwhile, *Porticoccus* was strongly positively correlated with innate immune genes *lectp1* and *lectp2* in AL × AL offspring and similarly (though not significantly) in IF × IF, but not in offspring from mixed-diet crosses. Likewise, *Dokdonia* showed positive associations with immune markers *AIF* and *intf* in IF × IF, AL × AL, and (non-significantly) AL(p) × IF(m) offspring, but this association reversed in IF(p) × AL(m) offspring, in which the father experienced IF. This reversal could imply that paternal fasting, when not mirrored by the mother, disrupts coordinated immune-microbiome interactions, potentially altering the offspring’s gut environment. Of course, we cannot exclude that microbial colonization during later developmental stages or environmental exposure may further shape these patterns. In a species like *S. typhle*, where both mothers and brooding fathers contribute to the microbial inoculum — via eggs and brood pouch environments — matched parental diets may support the maintenance or co-transfer of a similar microbial community [49, 84]. Furthermore, when diets match, parental epigenetic and physiological cues (e.g., immune tolerance, gut pH/mucosal environment, antimicrobial peptides) may be aligned, producing a gut environment in the offspring that selects for a consistent microbial community. With mismatched diets, the offspring may receive conflicting cues — say, maternal tolerance toward one microbial profile and paternal priming toward another — leading to disrupted or restructured microbiome assembly.

## Conclusions

Our study revealed how parental nutritional shifts shape offspring phenotype through sex-specific and asymmetric pathways in the sex-role-reversed pipefish *Syngnathus typhle*. By manipulating maternal and paternal diets, we demonstrate that maternal provisioning plays a dominant role in determining offspring condition, while paternal effects emerge more clearly in immune gene expression and microbiota composition. This highlights the importance of considering both maternal and paternal contributions — especially in systems where traditional sex roles are decoupled.

Notably, females experienced a greater decline in the condition index under IF, which translated into lower offspring condition, regardless of paternal diet. In contrast, offspring of IF males and well-fed females showed no such deficit, underlining the critical role of maternal provisioning in early development. However, IF fathers did not remain passive contributors; they exhibited strong immune activation and altered microbiomes, both of which were reflected in offspring gene expression and microbial profiles. These findings suggest that paternal nutritional stress can prime offspring immune function, likely through epigenetic and microbial inheritance mechanisms. Importantly, mismatched parental diets disrupted expected immune–microbiome correlations in offspring, indicating that coordinated parental cues may be essential for stable microbial and physiological development. Such interactions between diet, immunity, and microbiota underscore the ecological relevance of parental environmental history in shaping offspring resilience.

This research leverages the unique reproductive biology of *Syngnathus typhle* to disentangle the often-confounded effects of maternal provisioning and gestational environment under nutritional stress. It contributes a unique perspective to the study of intergenerational plasticity by moving beyond conventional reproductive models. Understanding these dynamics is critical in an era of rapid ecological change, where nutritional stress and altered resource availability are increasingly common due to climate change. *S. typhle* provides a powerful model for investigating how sex-specific and gestational pathways interact to mediate offspring outcomes across generations.

## Methods

### Study species and collection area

*Syngnathus typhle* were caught in the southwest Baltic Sea on the island Fehrman at the bay of Orth (54° 27′ N, 11° 3′ O) in late April 2022, using snorkelling gear and hand nets. The broadnosed pipefish were then transported back to Kiel University in appropriate containers connected to air stones and filled with Baltic Sea water from the field. At the institute, they were divided into sex-aggregated quarantine tanks maintained at 18 ppt salinity and 12 °C. To prevent the introduction of harmful parasites into the aquaria, the fish received three 1-h anti-parasite treatments using a 37% formalin solution at a 1:8000 concentration. Subsequently, the fish were allocated to sex-disaggregated 100-l tanks (50 L × 40 W × 51 D), where they were gradually acclimated over 2 weeks to their new environment. During this period, they were transitioned from live food to frozen mysids. The final water temperature was maintained at 18 °C, and fish were kept under a simulated day-night light cycle (i.e., 14 L:10 D).

### Experimental design

Pipefish were tagged with elastomer implants in red, blue, or orange by lightly sedating them with 0.02% tricaine mesylate (MS-222, Sigma-Aldrich). Tags were injected subcutaneously using a syringe on the left side of the body (Additional file 1: Fig. S1). During this brief procedure, the fish were also measured for size and weight.

The tagged fish were then sorted into groups of three (one red, one blue, and one orange), separated by sex, and housed in 28 aquaria. Each aquarium was randomly assigned to either the ad libitum (AL) food treatment or the intermittent fasting (IF) treatment (total *n* = 84: 21 AL males, 21 AL females, 21 IF males, 21 IF females). Fish in the AL treatment were fed defrosted mysids twice daily (8 a.m. and 4 p.m.), while those in the IF treatment were fed every third day, leading to two consecutive days of fasting. Given that *Syngnathus typhle* lack stomachs [85], overfeeding was unlikely; thus, the fasting group experienced a caloric reduction. To ensure observed effects were due to fasting and not nutrient deficiencies, both groups received enriched mysids every 6th day. The food was supplemented with multivitamins (15 µL of JBL Atvitol) and fatty acids (Omega-3 1000 capsules) and were allowed to absorb into the thawed mysids for 2–3 min. The feeding regime lasted 30 days, during which the water temperature was gradually increased by 1 °C every 5 days to reach 18 °C, simulating the natural conditions of the Baltic Sea known to trigger optimal mating behavior in pipefish.

After the monthlong treatment, the pipefish were removed from their respective aquaria and paired for mating, creating the following crosses (each replicated seven times): AL(p) × AL (m), IF(p) × IF(m), AL(p) × IF(m), and IF(p) × AL(m). A mating window of 24–48 h was provided, which was typically sufficient for successful mating, as both sexes had been separated for a month and were primed to mate at 18 °C. Pregnant males were returned to their treatment tanks, while females were euthanized with an overdose of MS-222 (500 mg/L), measured for total body length and weight, and dissected for further analysis (Additional file 3). The pregnant males were kept on their AL or IF diet, and approximately 1 month later, they gave birth. At the time of birth, five offspring (or as many as were present) were collected from each male. After male parturition, fathers and their offspring were euthanized, measured, and dissected. The initial tagging and tank assignments allowed for accurate matching of each fish to its initial size and weight measurements.

### Sample collection

After euthanasia, parental pipefish were dissected to remove the head kidney and liver, which were placed into 2-mL Eppendorf tubes containing RNAlater. These samples were stored in a refrigerator for 2 days and then transferred to − 20 °C awaiting further analysis. The intestinal tract was also removed, and a 1-cm section from the aboral part was placed into a sterile 1.5-mL Eppendorf tube using sterile tools. This sample was flash-frozen and stored at − 80 °C. All remaining gut tissue and gonads were collected for fat analysis.

Due to their small size, offspring were measured using a transparent container positioned over the millimeter paper, and images were analyzed for size using ImageJ (Version 1.53t; Schneider, Rasband, and Eliceiri, 2012) (Additional file 1: Fig. S2). Offspring were then euthanized and weighed using a precision scale. The gut was delicately extracted and placed into individual PCR tubes, which were stored at − 80 °C. The remaining body was submerged in RNAlater and processed like the adult tissue samples.

### Fat content measurement

Parent fat content was measured following the methods described by Auer (2010) [86]. The tissues were dried in an oven for 2 days at 60 °C and weighed using a precision scale. Samples were then submerged in anhydrous diethyl ether to dissolve triglycerides for 30 min, after which the ether (containing the dissolved fat) was discarded. The samples were air-dried for 1 h and weighed again. This process was repeated until the samples reached a constant lean dry weight. The total fat content was then measured by subtracting the initial weight with the final dried weight (Additional file 3).

### RNA extraction, reverse transcription, and gene expression

We quantified the mRNA levels of 44 preselected target genes based on previous studies [9, 60, 61] using quantitative real-time polymerase chain reaction (qRT-PCR). This was done on a 96.96 dynamic array BioMark HD system (GE chip; Fluidigm, South San Francisco, CA, USA) as described by Beemelmanns and Roth (2016) [9]. The selected genes are listed in Table 1 and the supplementary material Additional file 4. These genes were grouped into functional categories, following previous literature: (i) innate immune system (immediate, nonspecific immune defense, e.g., phagocytosis), (ii) adaptive immune system (specific, antibody-mediated immune defense), (iii) innate and adaptive immune genes (genes involved in both pathways), (iv) complement system (supporting antibody and phagocytic cell-mediated responses), and (v) epigenetic modulators (genes involved in DNA methylation, histone de/methylation, and histone de/acetylation) (Additional file 4).

**Table 1:**
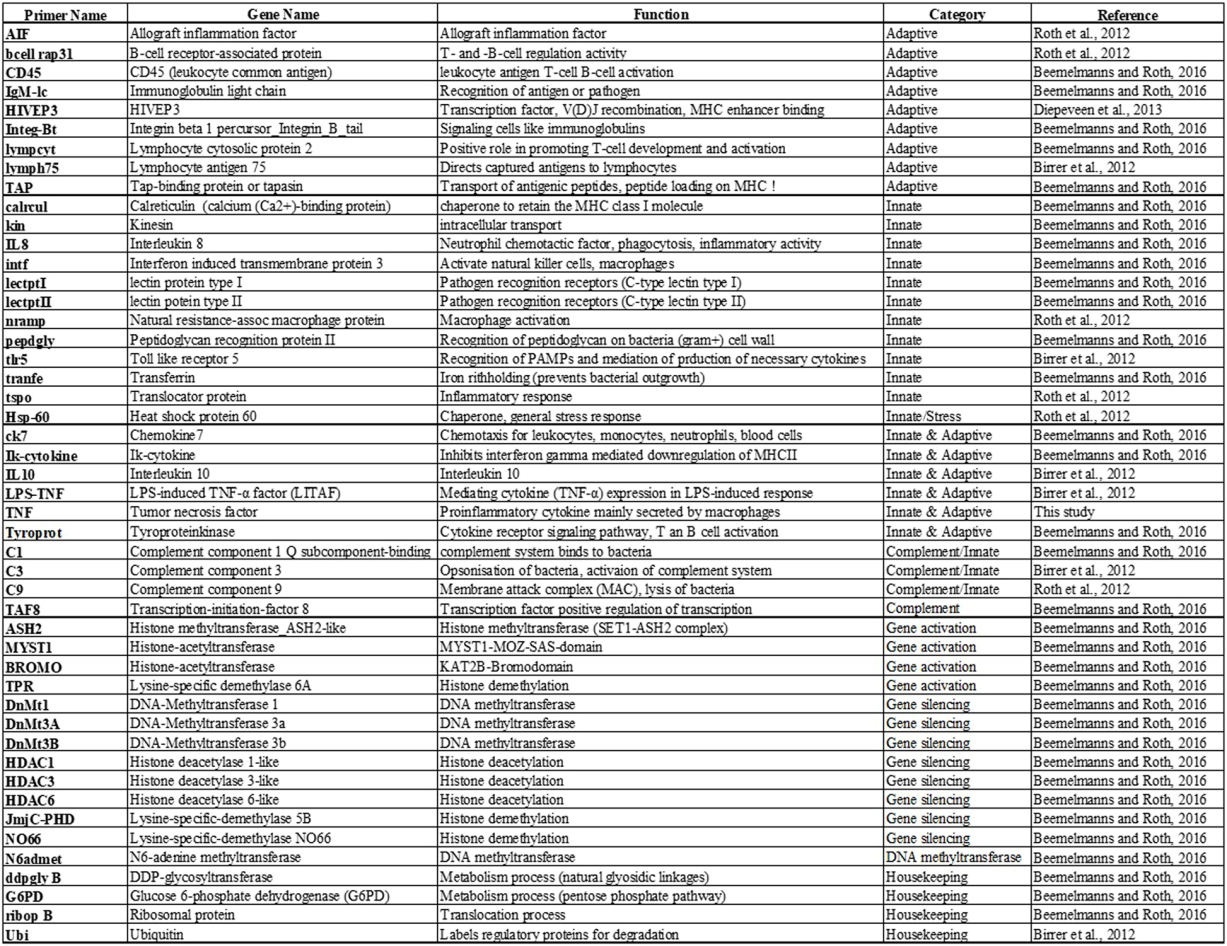
Name and function of 44 target genes and 4 housekeeping genes. Listed are all genes from different functional categories: adaptive, innate, innate and adaptive, complement system, gene activation, and gene silencing

RNA was extracted from the head kidneys of successfully mated males and females, as well as from whole-body tissue samples of five offspring from each parent, using the RNeasy Mini Kit (Qiagen, Venlo, Netherlands). The RNA concentration was measured with a DeNovix DS-11 Nanodrop, and all samples were normalized to 50 ng/µL before being stored at − 80 °C. cDNA synthesis was performed using the RevertAid First-Strand cDNA Synthesis Kit (Thermo Scientific, K1621) according to the manufacturer’s protocol, and the cDNA was stored again at − 80 °C for later use.

A pre-amplification step was conducted by mixing 1 μL of each of the 48 primers (500 nM, forward and reverse) into a primer pool. The reaction mixture consisted of 264 μL of 2 × TaqMan PreAmp Master Mix (Applied Biosystems, Waltham, MA, USA), 52.8 μL of primer pool mix, and 79.2 μL of purified water. Then, 3.8 μL of the mixture was combined with 1.3 μL of the cDNA sample. A dilution series (1:10, 1:20, 1:40, 1:80, 1:160, 1:320) was prepared to validate primer efficiencies (Additional file 4). The PCR settings were as follows: 95 °C for 10 min, followed by 14 cycles of 95 °C for 15 s and 60 °C for 4 min. The resulting PCR products were diluted 1:10 with low EDTA-TE buffer (10-mM Tris, 0.1-mM EDTA, pH 8) [87].

For the sample premix, we combined 369.6 μL of Ssofast-EvaGreen Supermix with Low ROX (Bio-Rad Laboratories, Hercules, CA, USA) and 37 μL of 20 × DNA Binding Dye Sample & Assay Loading Reagent (Fluidigm). Each well of the 96-well plate was loaded with 3.9 μL of this sample premix, followed by 3.1 μL of the diluted pre-amplified PCR products (in duplicate). The assay mix for the chip consisted of 369.6 μL of Assay Loading Reagent (Fluidigm) and 295.7 μL of low EDTA-TE buffer. A total of 6.3 μL of the assay mix was loaded into each well of a 96-well plate, and 0.7 μL of a 50-uM primer pair mix was added to each well (in duplicate). Finally, the GE chips were loaded with 5 μL of sample mix and 5 µL of assay mix and run on the BioMark system using the GE-fast 96.96 PCR + Melt v2.pcl protocol (Fluidigm). The plates included no-template controls (NTC), controls for gDNA contamination (NEG), and a serial dilution as mentioned previously.

### DNA extraction and sequencing

To extract microbiota from the adult hindgut and the entire offspring gut, DNA was isolated using the DNeasy Blood & Tissue Kit (QIAGEN, Germany), following the manufacturer’s protocol with modifications for gram-positive bacteria based on [88]. A 16S PCR was then performed to confirm successful extraction. Library preparation for a total of 114 samples (including 20 AL(p) × AL(m), 15 AL(p) × IF(m), 20 IF(p) × AL(m), 25 IF(p) × IF(m) offspring, along with 8 AL and 9 IF adult males, and 9 AL and 8 IF adult females) was completed by the Institute for Experimental Medicine (UKSH, Campus Kiel). Sequencing of the V3–V4 hypervariable region (341f/806r) was carried out using the Illumina MiSeq platform (Illumina, USA) with 2 × 300-bp paired-end settings at IKMB Kiel.

### Morphological and fat content data analysis

Statistical analyses were conducted using RStudio (Version 2024.04.2). We assessed changes in total body length (cm) and weight (g) before and after treatment for both male and female pipefish. Normality of the data was evaluated using the Shapiro–Wilk test, and given the small sample sizes, we also tested for homogeneity of variance with Levene’s test (Additional file 2). To assess changes in body size over time (pre- and post-treatment), we used linear mixed-effects models, accounting for individual variation (e.g., lmer(Body_Length ~ time × treatment + sex + (1 | ID)); Additional file 2: Sheet A). This approach was also applied to the weight data. Since sex could introduce confounding effects, especially considering that females only underwent 1 month of treatment compared to 2 months for males, we also analyzed males and females separately. For these analyses, we performed one-way ANOVAs on initial and final length and weight, using treatment as a factor (e.g., aov(Start_length ~ treatment; Additional file 2: Sheet A). Additionally, we calculated Fulton’s condition factor (weight/length^3^) to better assess overall fish condition [89], to which the same statistics were applied (Additional file 2: Sheet A). The fat body content was also measured for normality and variance distribution. After which a Kruskal–Wallis rank sum test with Benjamin-Hochberg correction was done for treatment and sex (Additional file 2: Sheet A). To test crossed parental dietary treatment effect for offspring size and weight, we used a one-way ANOVA (e.g., weight ~ crosstreatment), and if significant, we followed up with a Tukey’s HSD test (Additional file 2: Sheet A).

### Differential gene expression analysis

The quality of the chip run output was assessed by reviewing the Chip Run Info (Additional file 5). Samples flagged as problematic, and negative controls, were removed prior to further analysis. Each sample was analyzed in two technical replicates, and the mean cycle threshold (Ct) value was calculated for each sample. Primer efficiency values were determined from a dilution series for all primers (1:10, 1:20,1:40, 1:80, 1:160, 1:320). Any primer with efficiency below 80% was excluded from further analysis, including primers for the following genes: *C3*, *C9*, *Kin*, *ASH2*, *Ck7*, *N6admet*, *TAF8*, *Tyroprot*, and *tlr5* (Additional file 4). The geometric mean of Ct values for housekeeping genes (*ddpgly.B*, *G6PD*, *ribop.B*, and *Ubi*) was used to quantify relative gene expression for each target gene, calculated as ΔCt values (*Δ*Ct = mean Ct of target gene-geometric mean Ct of housekeeping genes). Samples or genes with excessive missing values were also excluded (i.e., *TNF*, with 25 NA values, Additional file 5). This cleaning process resulted in a final dataset comprising 34 genes and 104 samples (i.e., two plate chips), including 19 offspring from AL(p) × AL(m), 22 offspring from IF(p) × IF(m), 16 offspring from AL(p) × IF(m), and 17 offspring from IF(p) × AL(m) treatments.

A principal component analysis (PCA) was conducted to investigate differential gene expression profiles across parental dietary treatments (using the prcomp function in R). Additionally, a PERMANOVA model (utilizing the adonis2 function in the vegan package, e.g., adonis2(pca$x[,1] ~ treatment × family × plate, permutations = 999, method = “bray”); Additional file 2: Sheet B) was applied based on a Bray–Curtis distance matrix of the principal components. Separate PCA analyses were conducted for parent-only and offspring-only datasets to examine treatment effects independently for mothers, fathers, and offspring combinations (Additional file 2: Sheet B). Ellipses represent 80% confidence intervals. To assess treatment effects on individual genes, linear mixed models (LMMs) were applied with plate included as a random effect (lmer(gene ~ treatment + (1|plate), *lme4* package, Additional file 2: Sheet C). Family was included in the PCA PERMANOVA model but was not significant. Consequently, family was excluded from the final LMMs to avoid overfitting and model instability. This analysis was performed separately for males, females, and offspring. Genes that reached statistical significance were then mapped onto the PCA plot with their respective loadings. In this PCA visualization, arrow directions indicate gene correlations with the principal components, while arrow lengths represent each gene’s proportional contribution to overall variance. To calculate relative fold changes (LogFC), the ΔΔCt method was employed, representing the difference between the ΔCt values of the treatment and control groups. For parental samples (mothers and fathers), ΔΔCt was calculated as ΔCt_IF–ΔCt_AL. For offspring, ΔΔCt values compared combinations of parental treatments (ΔCt_IF(p) × AL(m)–ΔCt_AL(p) × AL(m)). Relative gene expression was calculated as 2^(-ΔΔCt) and then log2-transformed to normalize the distribution (Additional file 2: Sheet D). Interaction plots were created to illustrate gene expression patterns as influenced by maternal or paternal diet. These plots display mean ΔCt values (multiplied by − 1 for directionality) with error bars representing the standard error.

### Microbiome data analysis

The 16S rRNA amplicon sequencing analysis was carried out on the QIIME2 platform (v.2022.8.3) [90]. Using dada2, raw paired-end Illumina reads were initially demultiplexed, with primers for the 16S V3–V4 region then trimmed. Quality control involved denoising to distinguish genuine sequence diversity from sequencing errors. Further filtering removed low-quality reads (trimming forward reads to 260 bp and reverse reads to 210 bp). Forward and reverse reads were merged, and chimeric sequences were removed. A phylogenetic tree was constructed using FastTree (v.2.1.11) based on approximately maximum likelihood from the longest root [91]. Interactive α rarefaction curves were generated at a max depth of 20,000, assessing if sequencing depth was sufficient to capture the full bacterial community. Taxonomic classification for the V3–V4 hypervariable region was done with the SILVA v.138 database and a naive Bayes classifier [92]. Amplicon sequence variants (ASVs) were filtered to exclude chloroplast and mitochondrial sequences and were exported at the genus level as operational taxonomic units (OTUs) for further analysis in RStudio (v.2022.07.2).

The data was then filtered by removing singletons and applying a threshold of 0.2% of total reads per sample. After filtering, a total of 189 operational taxonomic units (OTUs) were retained for analysis. To better compare community composition across samples, we transformed the microbial count data to relative abundance. We analyzed community composition at the family and genus levels for parent and offspring treatment groups using *phyloseq* (v1.46.0). α-Diversity was measured with the Shannon and Simpson indices (vegan v2.6.4), and dietary treatment effects were assessed with the Kruskal–Wallis test and Dunn’s pairwise comparisons (Additional file 2: Sheet E). *β*-Diversity was evaluated using the Bray–Curtis dissimilarity matrix (abundance based) via the *vegdist* function, and hypothesis testing was conducted with PERMANOVA (Additional file 2: Sheet F). *β*-Diversity results were visualized with a nonmetric multidimensional scaling (NMDS). The NMDS ordination was used to visualize the dissimilarity between samples, with MDS1 and MDS2 representing the main sources of variation in community composition. In addition, phylogenetic *β*-diversity was assessed using weighted and unweighted UniFrac distance metrics, and results are provided in Additional file 1: Fig. S6. We fitted environmental factors (diet treatments) to the NMDS ordination, using the *envfit* function, and assessed the strength of the associations between bacterial taxa and treatment group (Additional file 2: Sheet F). To visualize these results, a heatmap was constructed (Fig. 5b), highlighting the most significant taxa that drive the observed patterns (p < 0.01, Additional file 2: Sheet F). Additionally, we generated a barplot depicting the family-level community composition, focusing on the top 20 taxa with the highest relative abundance (Fig. 5c).

To investigate potential relationships between gut microbial composition and gene expression in offspring, we selected a subset of taxa that contributed most significantly to differences among the dietary combinations in offspring (Fig. 5b). A Spearman correlation analysis was then performed between these taxa and the most significantly differentially expressed genes identified in the gene expression analysis (Fig. 4a and Additional file 2: Sheet B). We aimed to shed light on how parental diet might simultaneously shape the gut microbiota and gene regulatory networks in offspring, to explore potential communication pathways. The results were visualized with a heatmap using the *Hmisc* package (Fig. 6).

## Supplementary Information

Additional file 1: Figure S1: Tagged female *S. typhle. *Figure S2: Offspring size measurement. Figure S3: Body length and weight of *S. typhle* parents. Figure S4. Principal Component Analysisof parental and offspring gene expression data obtained from Fluidigm analysis. Figure S5. Interaction plots of the most significantly differentially expressed genes in*S. typhle*offspring based on parental dietary treatments. Figure S6: Non-metric multidimensional scalingordination of gut microbial community β-diversity based onunweighted UniFrac andweighted UniFrac distance metrics.

Additional file 2: Morphology statistics. Gene expression analysis statistics. PCA gene expression analysis statistics. Log Fold Change for differential gene expression analysis. Alpha diversity. Beta diversity

Additional file 3: Parental morphological measurements, including weight, total body length, condition index, and fat content, for both males and females across all treatment groups. Offspring morphological measurements of 5 individuals per family, including weight, total body length, condition index, and fat content, right after birth

Additional file 4: Information on the primers used for the Fluidigm analysis. Efficiency results calculated from the dilution series. Primer efficiency range between 0.8 and 1.2 are acceptable

Additional file 5: Raw Fluidigm Ct values organized with samples in rowsand columns for gene names and metadata, including Family, Sex, Treatment Group, and Plate Run

Additional file 6: 16S rRNA sequencing count data at the genus level, used for downstream analysis

## Data Availability

More information can be found in the supplementary files, including information on methodology and additional figures. Additional file 1: Figures S1-S6 contains Fig. S1 – Tagged female *S. typhle*; Fig. S2 – Offspring size measurement; Fig. S3 – Body length and weight of *S. typhle* parents; Fig. S4 – Principal component analysis (PCA) of parental and offspring gene expression data obtained from Fluidigm analysis; Fig. S5 – Interaction plots of the most significantly differentially expressed genes in *S. typhle* offspring based on parental dietary treatments; Fig. S6 UniFrac analysis. Additional file 2 displays the full statistical results in several panel sheets for A) morphology, B) PCA specific permanova analysis, C) LMM of gene expression analysis, D) the LogFC results, E) results for the alpha and F) Bray–Curtis Beta diversity analysis. Additional file 3 contains morphological raw data on length, weight, fat content and condition index of parents and offspring of *S. typhle*. Additional file 4 contains information on the primers we used for the Fluidigm analysis and their efficiency values. Additional file 5 has the raw CT values from the Fluidigm analysis. Additional file 6 has the raw genus level count data, metadata and taxa information. The raw sequencing 16S rRNA-Seq and metadata used in this study are available from the National Center for Biotechnology Information (NCBI) Sequence Read Archive (SRA) under BioProject ID PRJNA1270975 (Pappert F. Parental fasting effects on offspring in *Syngnathus typhle*. NCBI Sequence Read Archive. https://identifiers.org/ncbi/bioproject:PRJNA1270975 (2026)).

## Abbreviations

AIF: Allograft inflammation factor
AL: Ad libitum
ASH2: Histone methyltransferase ASH2-like
BROMO: Histone acetyltransferase
B-cell rap: B-cell receptor-associated protein
C1: Complement component 1 Q subcomponent binding
C3: Complement component 3
C9: Complement component 9
calrcul: Calreticulin (calcium-binding protein)
CD45: Leukocyte common antigen
ck7: Chemokine 7
ddpgly B: DDP-glycosyltransferase
DnMt1: DNA methyltransferase 1
DnMt3A: DNA methyltransferase 3a
DnMt3B: DNA methyltransferase 3b
G6PD: Glucose-6-phosphate dehydrogenase
HDAC1: Histone deacetylase 1-like
HDAC3: Histone deacetylase 3-like
HDAC6: Histone deacetylase 6-like
HIVEP3: HIVEP3
Hsp-60: Heat shock protein 60
IF: Intermittent fasting
IgM-lc: Immunoglobulin light chain
Ik-cytokine: Ik-cytokine
IL8: Interleukin 8
IL10: Interleukin 10
intf: Interferon-induced transmembrane protein 3
Integ-Bt: Integrin beta-1 precursor (integrin β tail)
JmjC_PHD: Lysine-specific demethylase 5B
K: Fulton’s condition factor
kin: Kinesin
lectpt1: Lectin protein type I
lectpt2: Lectin protein type II
LMM: Linear mixed models
LogFC: Log-fold change
LPS-TNF: LPS-induced TNF-α factor (LITAF)
lympcyt: Lymphocyte cytosolic protein 2
lymph 75: Lymphocyte antigen 75
MYST1: Histone acetyltransferase
N6admet: N6-adenine methyltransferase
NMDS: Nonmetric multidimensional scaling
NO66: Lysine-specific demethylase NO66
nramp: Natural resistance-associated macrophage protein
OTU: Operational taxonomic unit
PCA: Principal component analysis
pepdgly: Peptidoglycan recognition protein 2
ribop B: Ribosomal protein
TAF8: Transcription initiation factor 8
TAP: Tap-binding protein (tapasin)
TGIP: Trans-generational immune priming
TGP: Trans-generational plasticity
tlr5: Toll-like receptor 5
TNF: Tumor necrosis factor
TPR: Lysine-specific demethylase 6A
tranfe: Transferrin
tspo: Translocator protein
Tyroprot: Tyrosine protein kinase
Ubi: Ubiquitin
WGP: Within-generational phenotypic plasticity
