# Supplementary material for: Parental fasting effects on offspring immune gene expression, epigenetic patterns, and gut microbiota in a species with male pregnancy (Syngnathus typhle)
Source: BMC Biol. 2026 Jan 14;24:24. doi: 10.1186/s12915-026-02509-7 (PMC12849595; doi:10.1186/s12915-026-02509-7)
Supplement: Supplementary file 1 — Additional file 1: Figure S1: Tagged female S. typhle. Figure S2: Offspring size measurement. Figure S3: Body length and weight of S. typhle parents. Figure S4. Principal Component Analysisof parental and offspring gene expression data obtained from Fluidigm analysis. Figure S5. Interaction plots of the most significantly differentially expressed genes inS. typhleoffspring based on parental dietary treatments. Figure S6: Non-metric multidimensional scalingordination of gut microbial community β-diversity based onunweighted UniFrac andweighted UniFrac distance metrics. [file 12915_2026_2509_MOESM1_ESM.docx]

**Additional file 1**

**Methods**


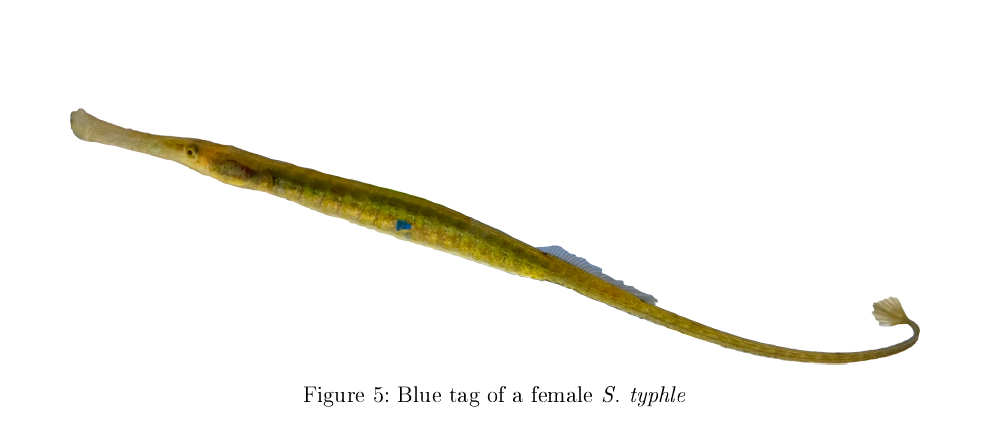


**Figure S1:** **Tagged female *S. typhle***

Example of a female *S. typhle* and the elastomer tagging.


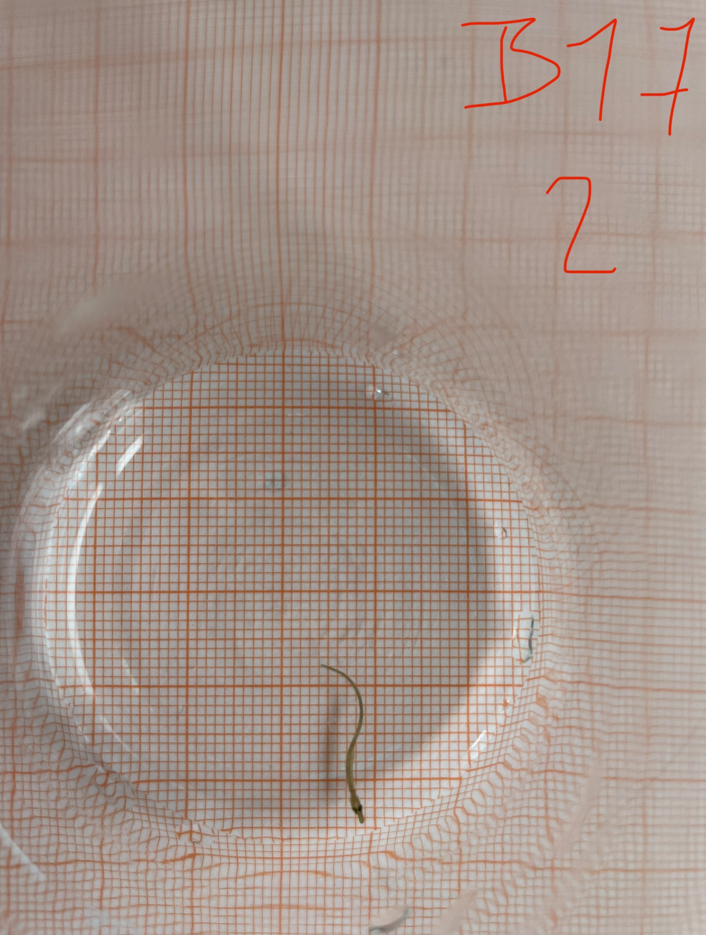


**Figure S2:** **Offspring size measurement**

Example of size measurement of offspring *S. typhle* after birth using millimetre paper and then ImageJ.

**Results**

**Intermittent fasting impacts on parental and offspring condition**

For AL males, body length increased significantly from the start with 11.7 cm ± 0.44 to the end of the two months diet with 13.6 cm ± 0.43 (p < 0.0001), as did AL females (p < 0.0001) starting at 12.97 cm ± 0.89 and growing to 13.91 cm ± 0.79 after one month. In contrast, IF-treated individuals exhibited more modest growth: IF males from 13.0 cm ± 0.76 reaching 14.1 cm ± 0.78, and IF females from 12.93 cm ± 0.93 to 14.08 cm ± 0.87 (both p < 0.02) (Additional file 2: Sheet A; Additional file 3).

The initial size of AL males was 0.68 g ± 0.07, increasing to 1.03 g ± 0.09 after two months. In comparison, IF males started at 0.94 g ± 0.14 and reached 1.06 g ± 0.17. Female AL individuals began at 1.02 g ± 0.24 and grew to 1.13 g ± 0.19 after one month, while IF females started at 0.99 g ± 0.27 and reached 0.92 cm ± 0.23.

a) b)


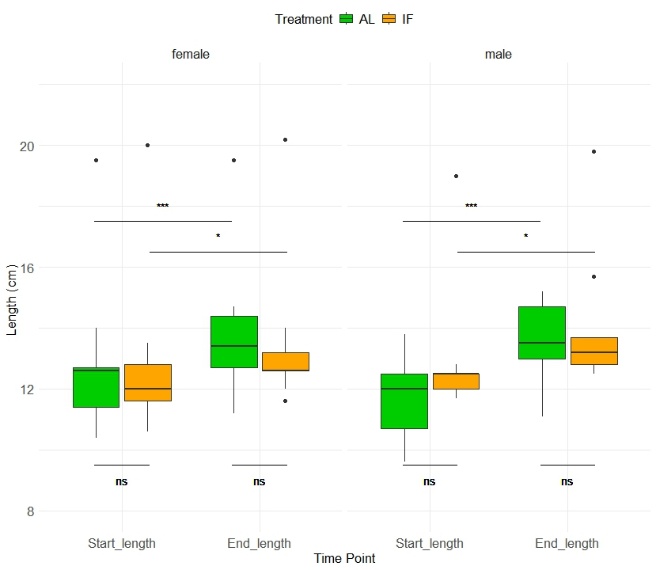

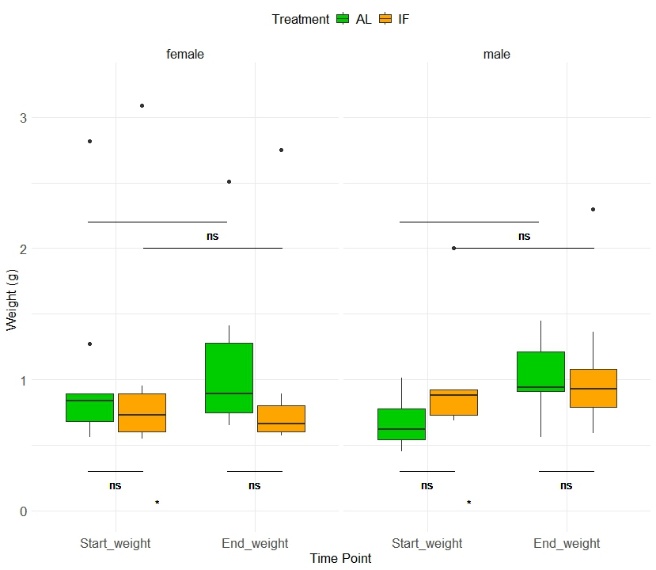


**Figure S3: Body length and weight of *S. typhle* parents**

a) The plot displays the body length of male and female pipefish before (Start_length) and after the treatment (End_length), with females shown on the left and males on the right. AL individuals are represented in green, and IF individuals are in orange. Two-way ANOVA results comparing treatment groups for both time points and sexes were not significant (ns). The linear mixed model results show significant changes in body size over time, with the IF group marked in orange (p < 0.02) and the AL group in green (p < 0.0001). b) In this barplot we see the body weight of male and female pipefish before (Start_length) and after the treatment (End_length). Two-way ANOVA results comparing treatment groups for both time points and sexes were not significant (ns). The linear mixed model results show significant changes in body weight over time, for AL (p = 0.014), but not for IF (p = 0.86).

**Intermittent fasting drives gene expression shifts in father’s and their offspring**

a) b)


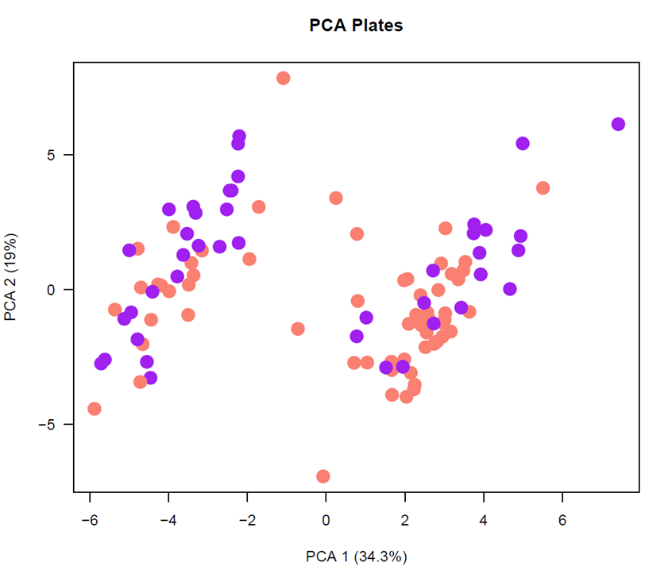

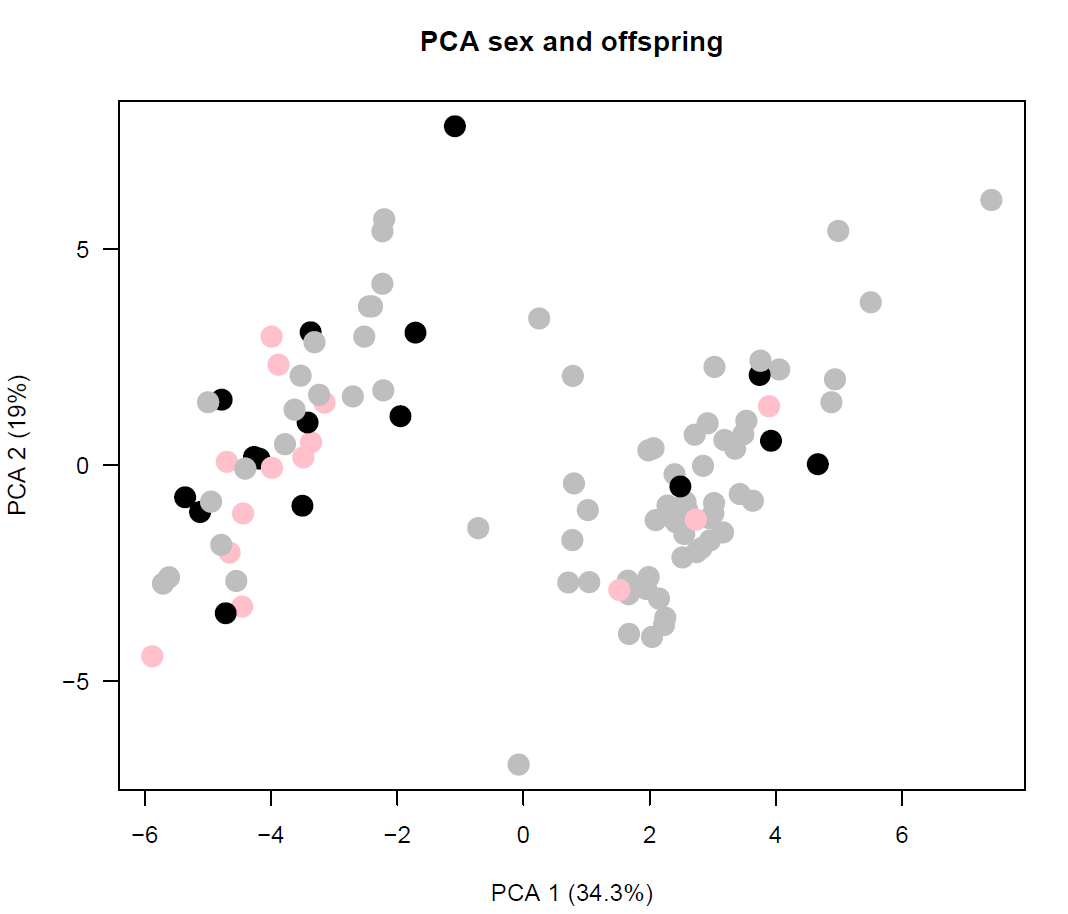


c)


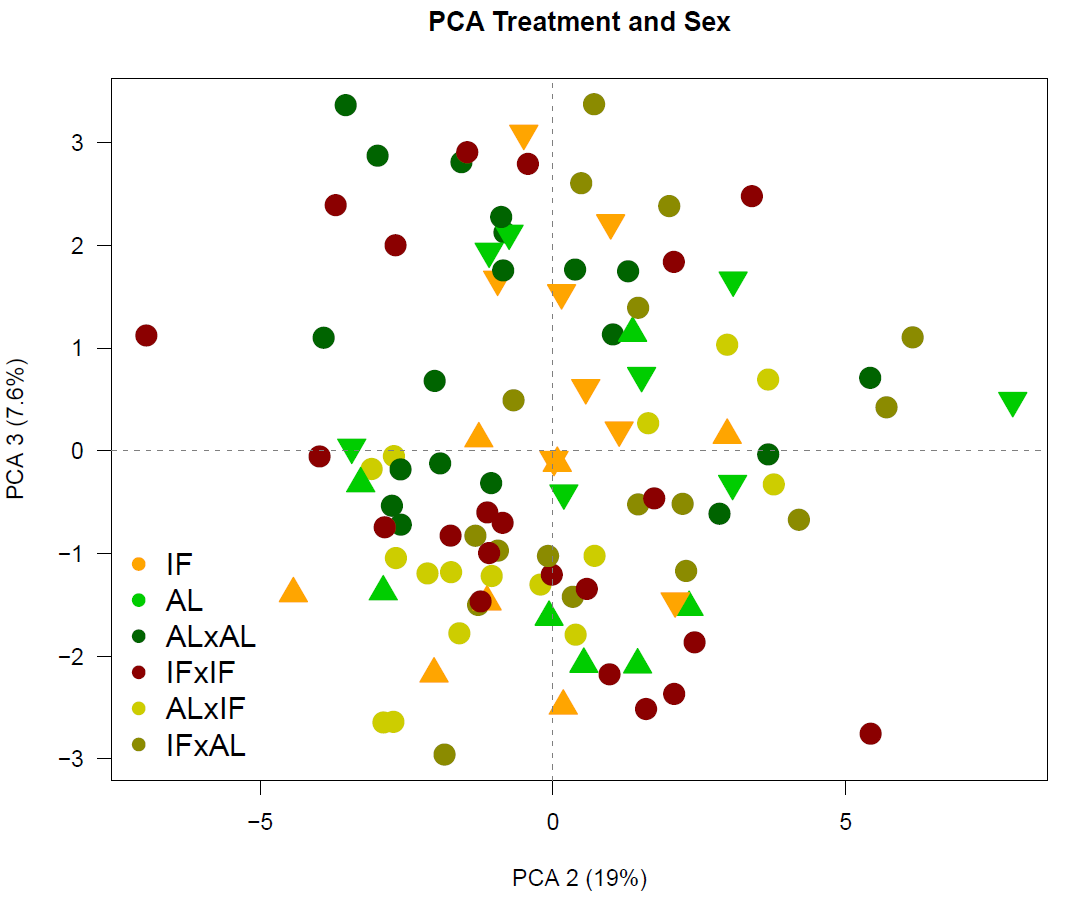


**Figure S4. Principal Component Analysis (PCA) of parental and offspring gene expression data obtained from Fluidigm analysis.**

Plots (a) and (b) represent PCA results for PC1 and PC2, with PC1 explaining 34.3% of the variance and PC2 accounting for 19%. In (a), dots are color-coded as purple or pink to differentiate between the two plate runs. A PERMANOVA on PC1 revealed a significant plate effect (p = 0.013), as well as a significant distinction between parents and offspring (p = 0.03). In (b), offspring data points are shown in gray, fathers in black, and mothers in light pink, indicating a trend for parental data to cluster towards the left of the PCA plot. Plot (c) illustrates the PCA for PC2 and PC3, with PC3 explaining 7.6% of the variance. Here, data points are color-coded by treatment group (dark green for AL(p)×AL(m), dark yellow for AL(p)×IF(m), brown for IF(p)×AL(m) and red for IF(p)×IF(m)), with triangles pointing down for females and up for males, distinguishing parental and offspring treatment groups. A PERMANOVA on PC3 showed a significant parent-offspring effect (p = 0.02) and a near-significant effect for treatment (p = 0.058).

a) b)


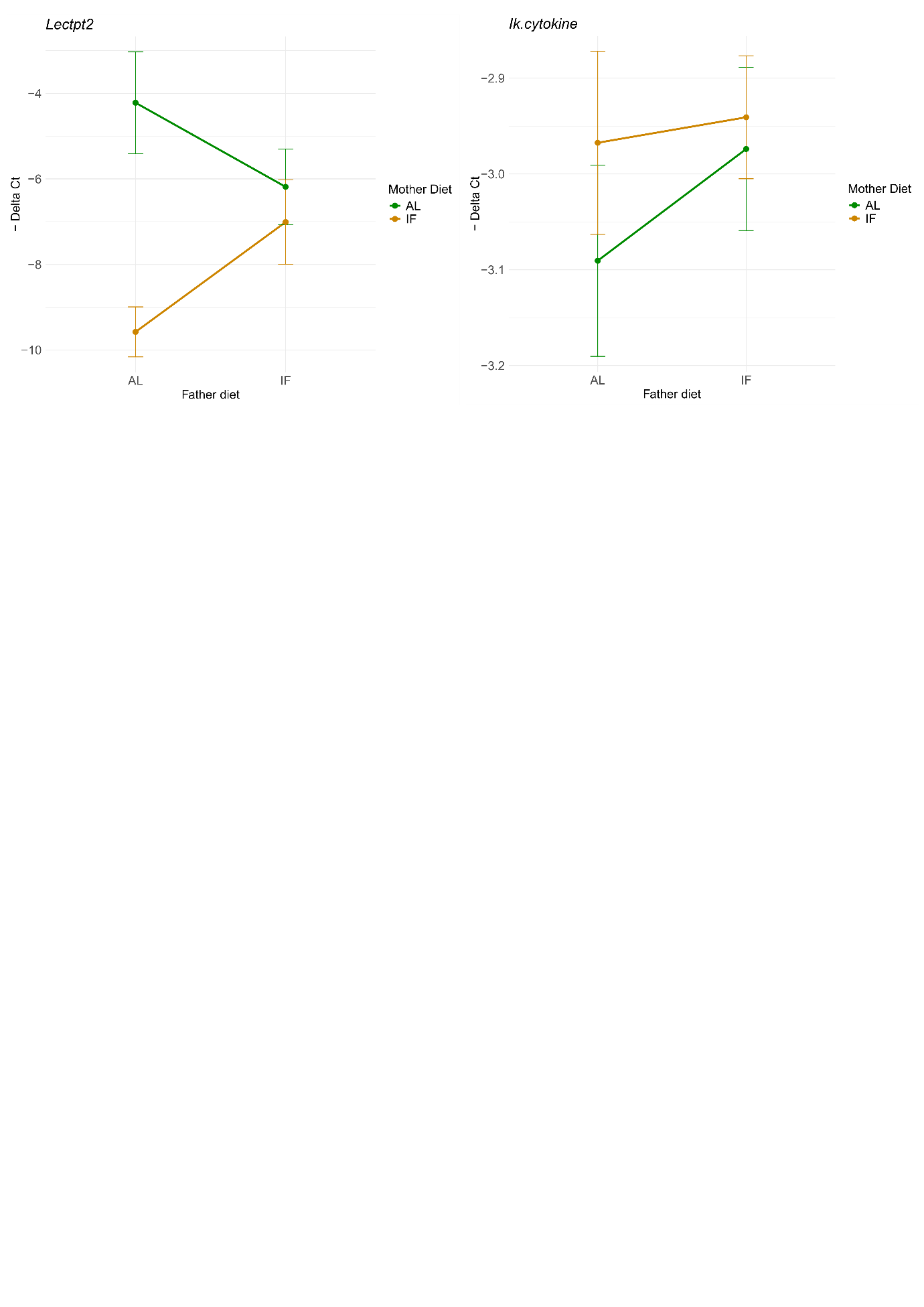


c) d)


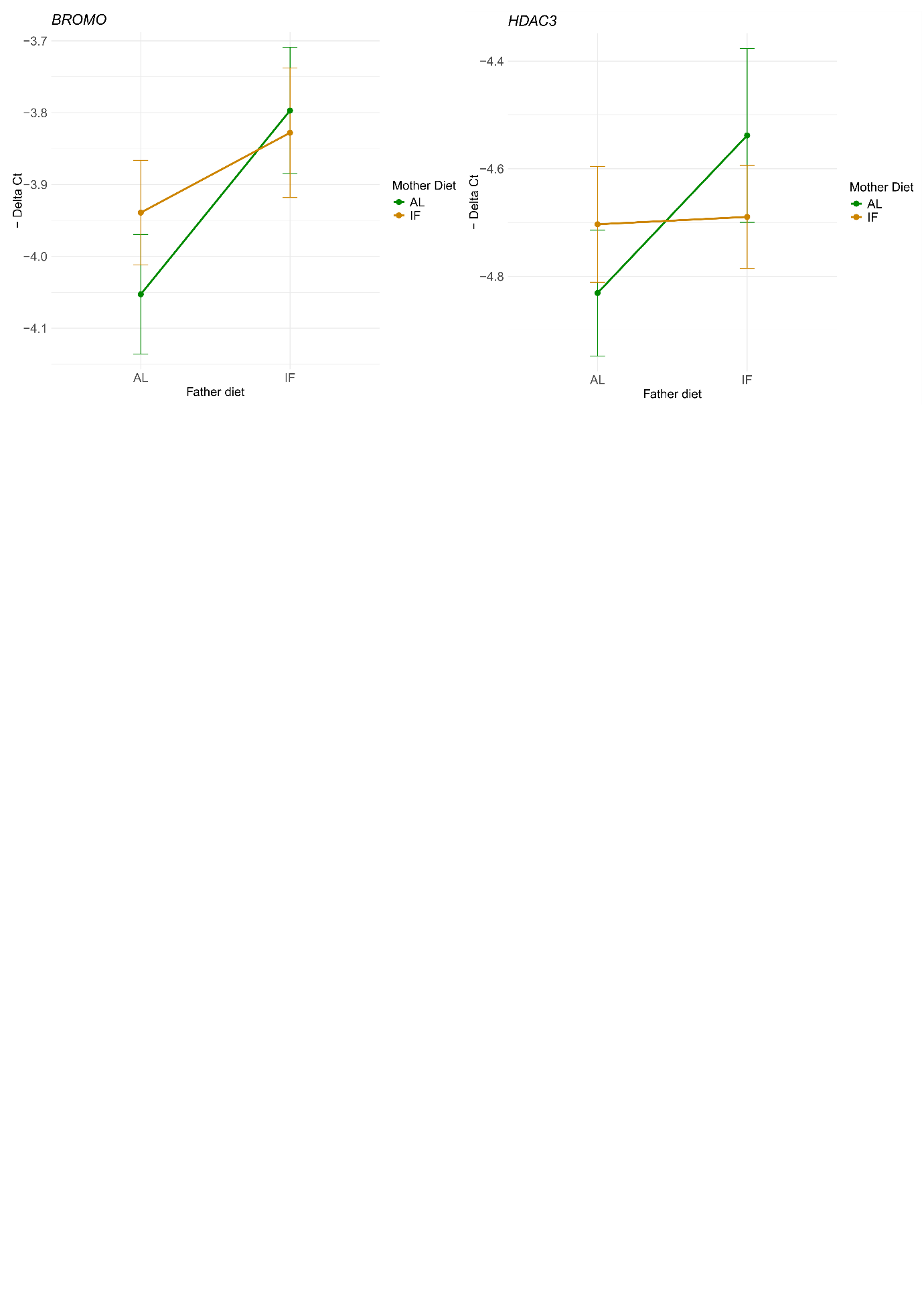


e) f)


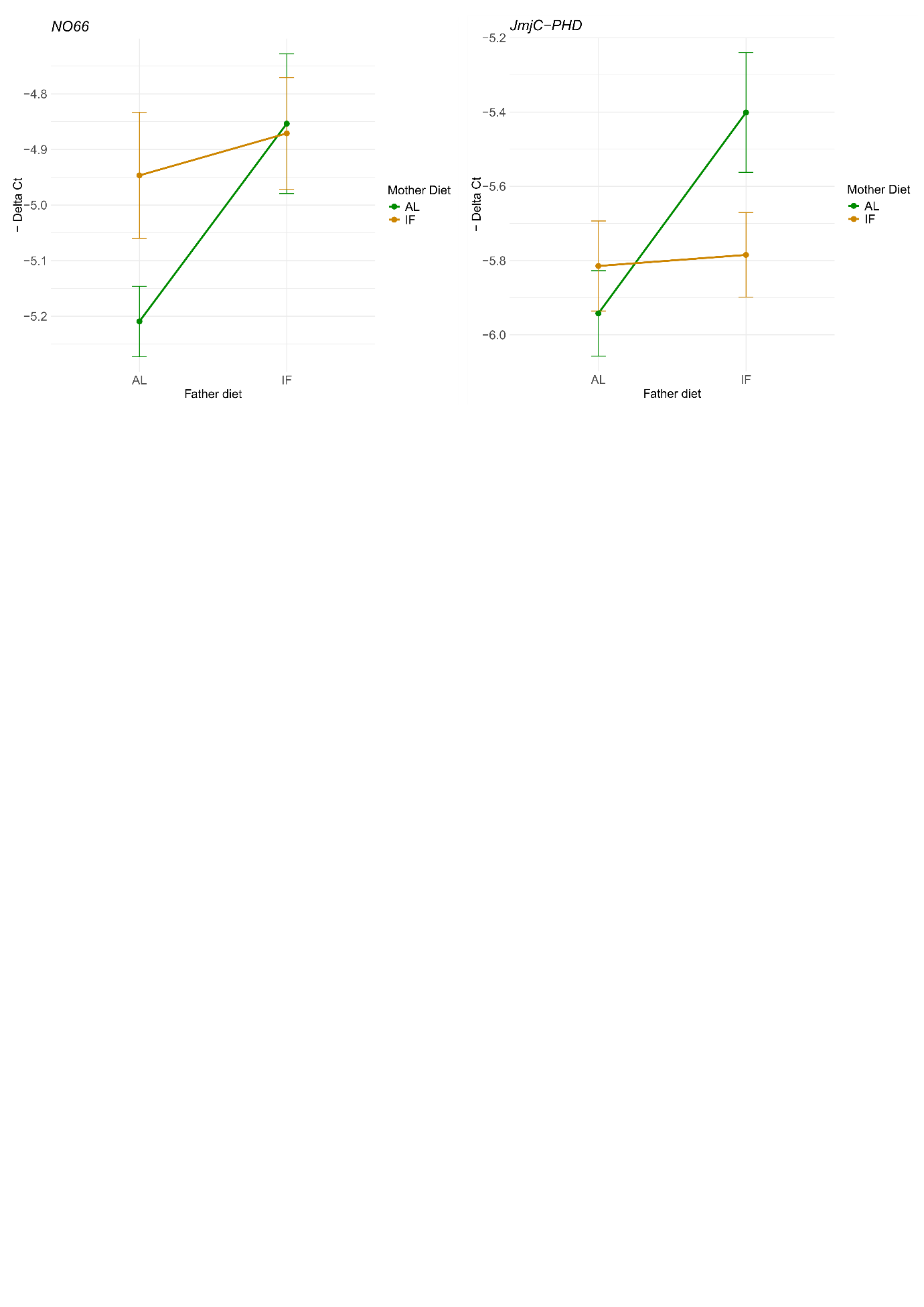


**Figure S5. Interaction plots of the most significantly differentially expressed genes in S. typhle offspring based on parental dietary treatments.**

Plots a) to (f) show interaction plots for significantly differentially expressed genes in *S. typhle* offspring, including *Lectpt2* (a), *Ik.cytokine* (b), *BROMO* (c), *HDAC3* (d), *NO66* (e), and *JmJC-PHD* (f). Additional genes, are found in the main manuscript. The x-axis represents the father’s diet, while colour coding (green for ad libitum and orange for intermittent fasting) indicates the mother’s diet. The y-axis displays the negative Delta Ct values, reflecting the directionality of gene expression. Each plot illustrates the interactions between parental dietary treatments and their effects on offspring gene expression.

**Fasting affects microbial composition of father’s and offspring**

a) b)


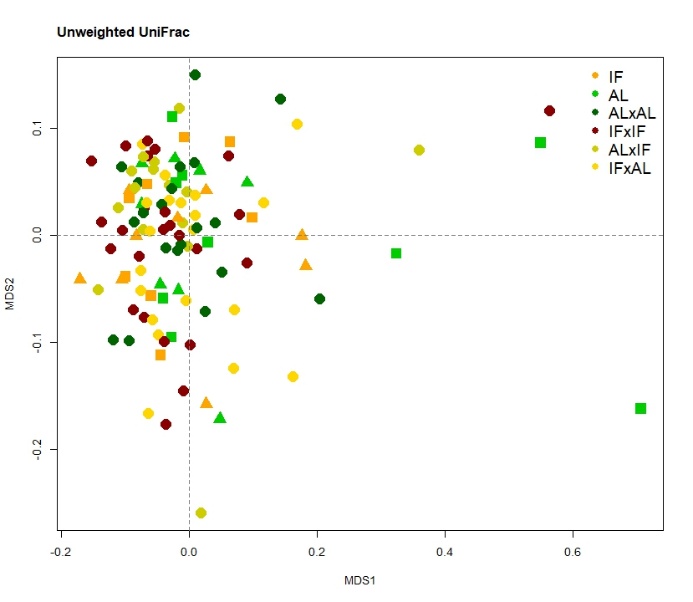

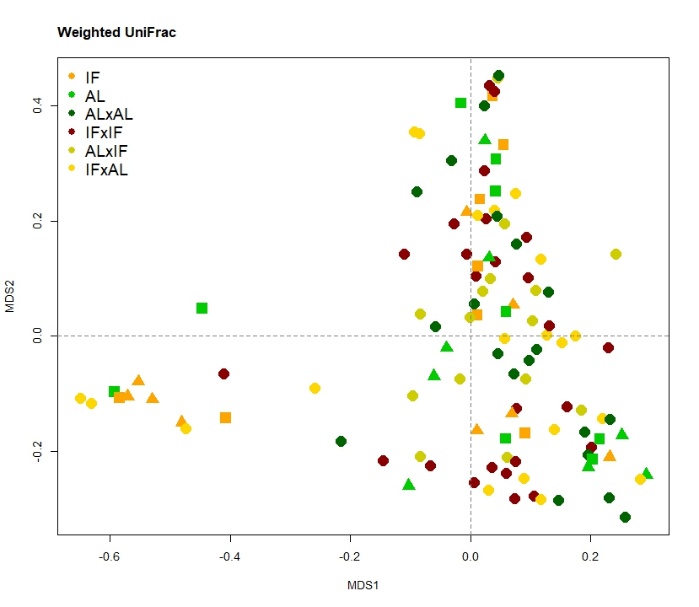


**Figure S6: Non-metric multidimensional scaling (NMDS) ordination of gut microbial community β-diversity based on (a) unweighted UniFrac and (b) weighted UniFrac distance metrics.**

UniFrac distances incorporate phylogenetic relationships among bacterial taxa, with a) unweighted UniFrac reflecting presence/absence of bacterial lineages and b) weighted UniFrac additionally accounting for their relative abundances. Each point represents one sample, and distances between points indicate dissimilarity in community composition. Points are coloured according to diet: fasting parents are shown in orange and ad libitum parents in light green. Offspring colour-coding reflects parental diet combinations, with dark green indicating offspring from two ad libitum parents, dark red offspring from two fasting parents, olive green offspring from an ad libitum male and fasting female, and yellow offspring from a fasting male and ad libitum female. Parental sex is denoted by symbols, with triangles representing males and squares representing females. The absence of clustering by generations or parental diet in UniFrac-based ordinations (p > 0.05, Additional file 2: Sheet F) indicates that differences in gut microbial communities are not driven by shifts in phylogenetic lineage composition, but rather by changes in the relative abundance of taxa within shared bacterial lineages.
